# SIRT1 overexpression ameliorates a mouse model of SOD1-linked amyotrophic lateral sclerosis via HSF1/HSP70i chaperone system

**DOI:** 10.1186/s13041-014-0062-1

**Published:** 2014-08-29

**Authors:** Seiji Watanabe, Natsumi Ageta-Ishihara, Shinji Nagatsu, Keizo Takao, Okiru Komine, Fumito Endo, Tsuyoshi Miyakawa, Hidemi Misawa, Ryosuke Takahashi, Makoto Kinoshita, Koji Yamanaka

**Affiliations:** 1 Department of Neuroscience and Pathobiology, Research Institute of Environmental Medicine, Nagoya University, Furo-cho, Chikus, Nagoya 464-8601, Japan; 2 Department of Molecular Biology, Division of Biological Sciences, Nagoya University Graduate School of Science, Furo-cho, Chikusa, Nagoya 464-8602, Japan; 3 Laboratory for Motor Neuron Disease, RIKEN Brain Science Institute, Wako, Saitama, Japan; 4 Department of Pharmacology, Faculty of Pharmacy, Keio University, Tokyo, Japan; 5 Center for Genetic Analysis of Behavior, National Institute for Physiological Sciences, Okazaki, Japan; 6 Genetic Engineering and Functional Genomics Group, Frontier Technology Center, Kyoto University Graduate School of Medicine, Kyoto, Japan; 7 Division of Systems Medical Science, Institute for Comprehensive Medical Science, Fujita Health University, Toyoake, Japan; 8 Department of Neurology, Kyoto University Graduate School of Medicine, Kyoto, Japan; 9 CREST (Core Research for Evolutionary Science and Technology), JST (Japan Science and Technology Agency), Kawaguchi, Japan

**Keywords:** Sirtuin 1 (SIRT1), Cu/Zn-superoxide dismutase (SOD1), Heat shock factor 1 (HSF1), Heat shock protein (HSP), Amyotrophic lateral sclerosis (ALS), Systematic behavioral screening

## Abstract

**Background:**

Dominant mutations in superoxide dismutase 1 (SOD1) cause degeneration of motor neurons in a subset of inherited amyotrophic lateral sclerosis (ALS). The pathogenetic process mediated by misfolded and/or aggregated mutant SOD1 polypeptides is hypothesized to be suppressed by protein refolding. This genetic study is aimed to test whether mutant SOD1-mediated ALS pathology recapitulated in mice could be alleviated by overexpressing a longevity-related deacetylase SIRT1 whose substrates include a transcription factor heat shock factor 1 (HSF1), the master regulator of the chaperone system.

**Results:**

We established a line of transgenic mice that chronically overexpress SIRT1 in the brain and spinal cord. While inducible HSP70 (HSP70i) was upregulated in the spinal cord of SIRT1 transgenic mice (PrP-Sirt1), no neurological and behavioral alterations were detected. To test hypothetical benefits of SIRT1 overexpression, we crossbred PrP-Sirt1 mice with two lines of ALS model mice: A high expression line that exhibits a severe phenotype (SOD1^G93A^-H) or a low expression line with a milder phenotype (SOD1^G93A^-L). The *Sirt1* transgene conferred longer lifespan without altering the time of symptomatic onset in SOD1^G93A^-L. Biochemical analysis of the spinal cord revealed that SIRT1 induced HSP70i expression through deacetylation of HSF1 and that SOD1^G93A^-L/PrP-Sirt1 double transgenic mice contained less insoluble SOD1 than SOD1^G93A^-L mice. Parallel experiments showed that *Sirt1* transgene could not rescue a more severe phenotype of SOD1^G93A^-H transgenic mice partly because their HSP70i level had peaked out.

**Conclusions:**

The genetic supplementation of SIRT1 can ameliorate a mutant SOD1-linked ALS mouse model partly through the activation of the HSF1/HSP70i chaperone system. Future studies shall include testing potential benefits of pharmacological enhancement of the deacetylation activity of SIRT1 after the onset of the symptom.

## Background

Pathological protein aggregation is a major hallmark of neurodegenerative diseases including amyotrophic lateral sclerosis (ALS), Parkinson’s disease, Alzheimer’s disease and polyglutamine diseases [1]. In ALS, approximately 10% is inherited and about 20% of the inherited ALS are caused by dominant mutations in the gene encoding Cu/Zn-superoxide dismutase (SOD1) [2]. Many of ALS causative SOD1 mutants retain the enzymatic activity for catalyzing superoxide anion to hydrogen peroxide and deletion of wild-type SOD1 from mice does not cause ALS phenotype, suggesting that SOD1 mutants provoke motor neuron degeneration through “gain of toxic function” mechanisms [3]. It has been suggested that altered conformations of mutant SOD1 proteins are linked to the toxicity [4]. Recent studies demonstrated that accumulation of misfolded SOD1 proteins is not only specific to SOD1-related familial ALS but also involved in a subset of sporadic ALS [5],[6].

Chaperone system is a major cytoprotective mechanism against proteotoxic stresses. Unfolded or misfolded proteins are restored to their proper conformations with the help of heat shock proteins (HSPs) including HSP110/105, constitutive and inducible HSP70s (HSC70, HSP70i). HSP110/105, HSP70i, and HSP40 directly bind to mutant SOD1 and facilitate the clearance of SOD1 through the ubiquitin-proteasome pathway [7]–[9], suggesting that HSPs are involved in the neuroprotective mechanisms against SOD1-mediated neurodegeneration. Indeed, HSP70i co-expression in motor neurons substantially inhibits the SOD1-aggregation and improves the cell viability [10]. Moreover, HSP70i is also effective against other neurodegenerative diseases, such as Parkinson’s disease [11] and Alzheimer’s disease [12]. These data suggest that the induction of HSPs is a promising therapeutic strategy for neurodegenerative diseases including ALS.

Upon exposure to proteotoxic stresses, heat shock factor 1 (HSF1) forms a trimer, translocates to the nucleus, binds to regulatory elements of DNA, and upregulates a set of genes including HSP70i whose products constitute the chaperone system [13]. Deacetylation of HSF1, which is mediated partly by a sirtuin family deacetylase SIRT1, prolongs its binding to the heat shock promoters [14]. Recent studies have demonstrated beneficial effects of resveratrol, which is considered to potentiate the sirtuin cascade, on SOD1-ALS models [15],[16]. Given the pleiotropic effect of resveratrol through non-sirtuin targets, the role of SIRT1 in motor neuron degeneration is to be examined. However, the effect of genetic supplementation of SIRT1 in ALS mice has never been directly tested.

On the basis of the above background, in this study we establish transgenic mouse lines that express SIRT1 in the central nervous system (CNS) under control of the murine prion promoter (PrP). We find that modest overexpression of SIRT1 alone does not cause neurological and behavioral alterations. To test the effects of SIRT1 overexpression, we crossbred the line with either of the two lines of transgenic mice which express a high and a low dose of the toxic SOD1^G93A^ polypeptide that recapitulate selective motor neuron degeneration. We find that SIRT1 overexpression consistently endows partial rescue effects with the milder ALS model with the lower dose of SOD1^G93A^. Biochemical analysis of the spinal cord extracts reveals upregulation of HSP70i and reduced aggregation of SOD1^G93A^ in the SOD1^G93A^/PrP-Sirt1 mice, corroborating the beneficial role of SIRT1 partly through activating the HSF1/HSP70i system.

## Results

### Generation of a prion promoter-driven *Sirt1* transgenic mouse

**Figure 1:**
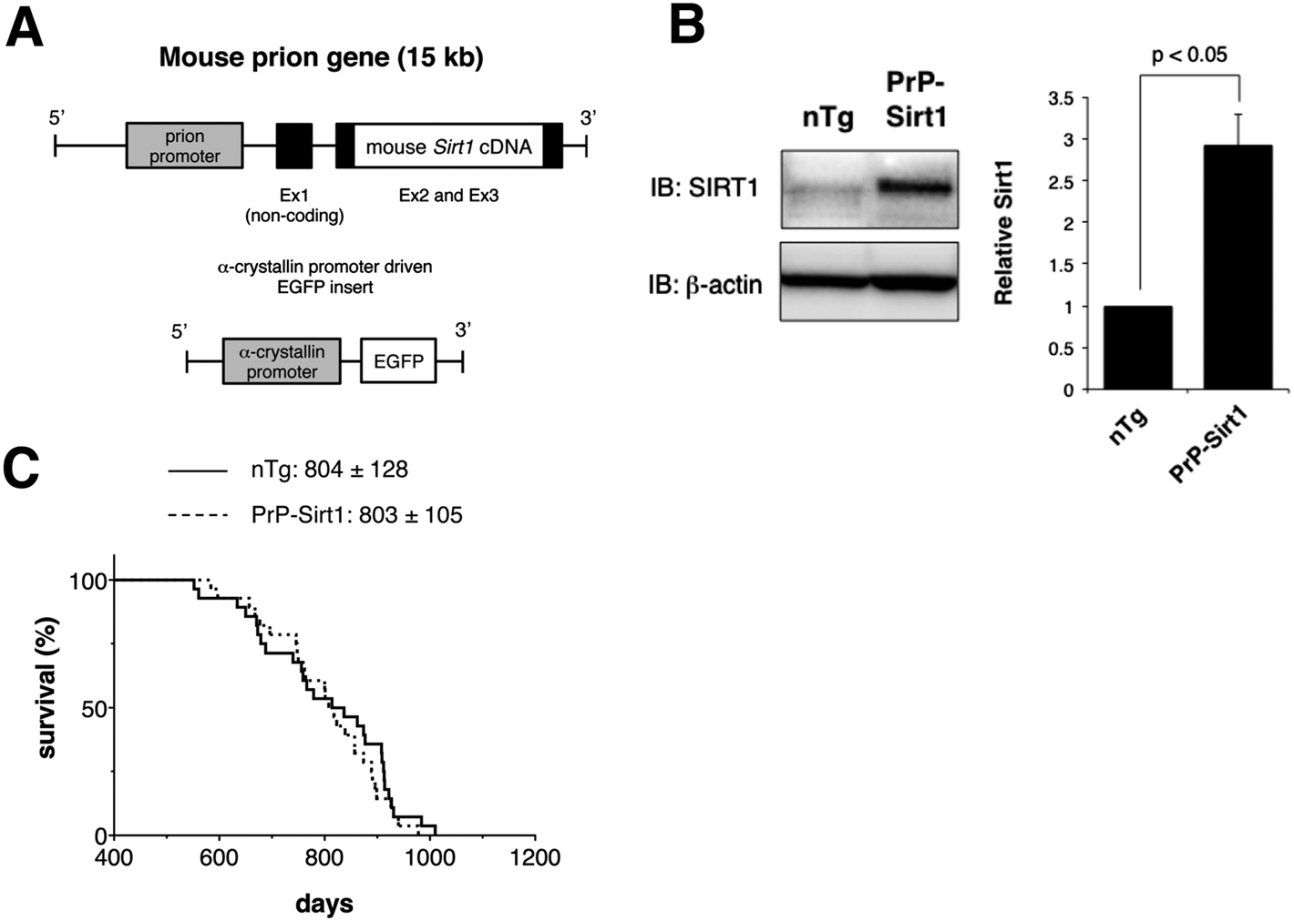
**Generation of a line of transgenic mice that overexpress SIRT1 (PrP-Sirt1). (A)** Schematic diagram of transgenes used to generate PrP-Sirt1 founder mice. Full-length mouse *Sirt1* cDNA was subcloned into the MoPrP (mouse prion promoter)-vector [37]. The non-coding portion of exon 2 and 3, and the *Sirt1* cDNA insert (with its own start and stop codon) were fused into one exon. The linearized transcription unit was co-injected with a visual marker, α-crystallin promoter-driven enhanced green fluorescent protein (EGFP) cDNA. **(B)** SIRT1 expression in PrP-Sirt1 and non-transgenic (nTg) mouse spinal cord. The expression level of Sirt1 was quantified in three independent experiments and shown as mean ± standard error of the mean (SEM). The data was analyzed by Student’s t-test. **(C)** Survival curve of the PrP-Sirt1 and nTg mice plotted over time (n = 28 each). Mean survival time (days) was shown with standard deviation (SD).

We generated transgenic mice that chronically express mouse SIRT1 in the CNS under control of the murine prion gene promoter (Figure 1A) (see Methods). The founder transgenic mice were backcrossed with wild-type C57BL/6 J mice, and a transgenic line with the highest SIRT1 protein level in the brain was selected. The offspring was further backcrossed for more than ten generations and established a transgenic line that gave consistent pan-neural expression of exogenous SIRT1. We compared the heterozygous *Sirt1* transgenic mice (PrP-Sirt1) with their non-transgenic littermates for the expression level of SIRT1 protein in the brain and spinal cord. Immunoblot analyses for SIRT1 confirmed the clear induction of active SIRT1 in the brain and spinal cord of PrP-Sirt1 mice (Figure 1B) (Additional file 1: Figure S1). In the PrP-Sirt1 mouse spinal cord, the expression level of SIRT1 protein was about three-fold higher than the one in the non-transgenic littermates. The lifespan of the PrP-Sirt1 mice was comparable to that of their non-transgenic littermates (Figure 1C).

### Chronic overexpression of SIRT1 in the CNS does not affect the behavior of mice

As a means of unbiased screening for potential neurological alterations, PrP-Sirt1 mice were subjected to a battery of physical and behavioral examinations. Overall, PrP-Sirt1 transgenic and non-transgenic male littermates (n = 15 and 20, tested at 5–9 months of age) did not differ in their physical and behavioral indices quantified, except for a few minor items (Table 1 and Additional file 1: Figure S3-S17. The raw data are accessible at the Mouse Phenotype Database, http://www.mouse-phenotype.org/). These findings indicate that chronic overexpression of SIRT1 in the mouse CNS does not cause major physical or neuropsychiatric alterations up to that age.

**Table 1:** Summary of systematic behavioral tests for PrP-Sirt1 mice in comparison with wild-type littermates

**Tests**	**Mental/physical activities**	**Indices measured**	**Alteration from wild type**
		**(inexhaustive)**	**PrP-Sirt1**
General health and neurological screening	General health	Body weight	↓
(Additional file 1: Figure S3)		Rectal temperature	→
		Grip strength	→
		Hanging persistence	↓
Light/dark transition test	Exploratory activity	Distance traveled in the light chamber	→
(Additional file 1: Figure S4)	Light avoidance	Distance traveled in the dark chamber	→
		Latency to the first entry to the light chamber	→
		Time stayed in the light chamber	→
		Number of transitions between chambers	↓
Open field test	Exploratory activity	Distance traveled	↑
(Additional file 1: Figure S5)	Avoidance from open space	Center time	→
	Anxiety-like behavior	Vertical activity	→
		Stereotypic counts	→
Elevated plus maze test	Exploratory activity	Distance traveled	→
(Additional file 1: Figure S6)	Height avoidance	Entries into open arms	→
		Number of entries	→
		Time stayed on open arms	→
Acoustic startle response	Startle reflex to loudness	Ampliture of body motion	→
(Additional file 1: Figure S7A)			
Prepulse inhibition (PPI) test	Sensorimotor gating	Decrement of startle amplitude	→
(Additional file 1: Figure S7B)			
Porsolt forced swim test	Despair-like behavior	Latency to immobility	→
(Additional file 1: Figure S8)			
Home cage monitoring	Diurnal cycle of locomotor activity	Activity level (distance traveled)	→
(Additional file 1: Figure S9)	Social behavior	Mean number of particles	→
Social interaction test	Social behavior, anxiety-like behavior	Distance traveled	→
(1 chamber, stranger pair)		Number of contacts	→
(Additional file 1: Figure S10)		Total duration of active contacts	→
		Mean contact duration	→
		Total duration of contacts	→
Social interaction test	Social behavior, anxiety-like behavior	Time spent with novel stranger	↓ (Step 2)
(3 chamber, 1–2 caged strangers)			
(Additional file 1: Figure S11)		Distance traveled	→
Rota-rod test	Motor coordination/learning	Latency to fall	→
(Additional file 1: Figure S12)			
Gait analysis	Mechanics of limb movement	Limb positions and timings	→ (front step angle)
(Additional file 1: Figure S13)			↑ (hind step angle)
Hot plate test	Aversive response to noxious stimulus	Latency to limb withdrawal	→
(Additional file 1: Figure S14)			
Tail suspension test	Behavioral despair	Latency to immobility	→
(Additional file 1: Figure S15)			
Barnes maze	Spatial memory	Time spent around each hole	→
(Additional file 1: Figure S16)			
Fear conditioning test	Fear memory	Conditioning	→
(Additional file 1: Figure S17)		Contextual testing	→
		Cued test with altered context	→

Comparative table of behavioral test results with PrP-Sirt1 and wild-type littermate C57BL/6 J mice. Symbols: →, no significant difference; ↑↓, statistically significant increase and decrease as compared with wild-type control mice. N.D., not determined.

### Endogenous SIRT1 is upregulated in the spinal cord of SOD1^G93A^ and SOD1^G37R^ transgenic mouse

**Figure 2:**
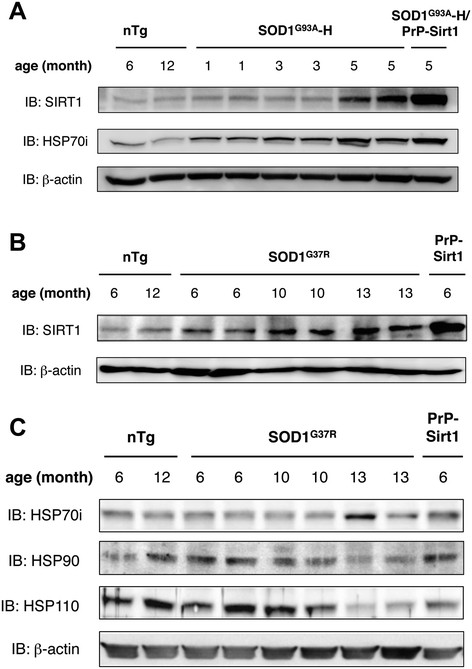
**Expression of endogenous SIRT1 and the major heat shock proteins in the spinal cord of SOD1**^**G93A**^**, SOD1**^**G37R**^**or SIRT1 transgenic mice. (A)** Endogenous SIRT1 and HSP70i were upregulated in high copy SOD1^G93A^ mouse (SOD1^G93A^-H) spinal cord. A representative immunoblot image for SIRT1 and HSP70i in the lumbar spinal cord of SOD1^G93A^-H, SOD1^G93A^-H and PrP-Sirt1 double transgenic (SOD1^G93A^-H/PrP-Sirt1), or non-transgenic (nTg) mice at designated age. Each lane contained 30 μg total protein and the similar results were obtained from three independent experiments. **(B)** Endogenous SIRT1 was upregulated in SOD1^G37R^ mouse spinal cord. A representative immunoblot image for SIRT1 in the lumbar spinal cord of SOD1^G37R^, PrP-Sirt1, or nTg mice at designated age. Each lane contained 20 μg total protein and the similar results were obtained from three independent experiments. **(C)** HSP70i was modestly induced in SOD1^G37R^ mouse spinal cord. Representative immunoblot images for HSP70i, HSP90, and HSP110 in the same lumber spinal cord homogenates as in **(B)**. Each lane contained 30 μg total protein and the similar results were obtained from three independent experiments.

We have previously reported that HSP70i is induced in the spinal cord of SOD1^G93A^ transgenic mouse [9]. To test the possible involvement of SIRT1 in the induction of HSP70i, we investigated the expression level of SIRT1 in the spinal cords of high expression line of SOD1^G93A^ mouse (SOD1^G93A^-H) and SOD1^G37R^ transgenic mouse which expresses human SOD1 gene carrying another ALS-linked mutation [17],[18]. SIRT1 and HSP70i were clearly upregulated toward the end stage (5 months of age) of SOD1^G93A^-H mice (Figure 2A). In SOD1^G93A^-H/PrP-Sirt1 double transgenic mice, the level of HSP70i was not different from that of SOD1^G93A^-H mice (Figure 2A) (Additional file 1: Figure S1), suggesting that the induction of HSP70i had peaked out. Similarly, SIRT1 was upregulated from around the symptomatic onset (10 months of age) to the end stage (13 months of age) in the spinal cords of SOD1^G37R^ mice (Figure 2B). HSP70i was induced only at the end stage, when HSP90 and HSP110/105 were downregulated (Figure 2C), consistent with our previous results on the expression of HSPs in SOD1^G93A^ mice [9].

### SIRT1 overexpression extends lifespan of the transgenic mouse expressing low copy of SOD1^G93A^

**Figure 3:**
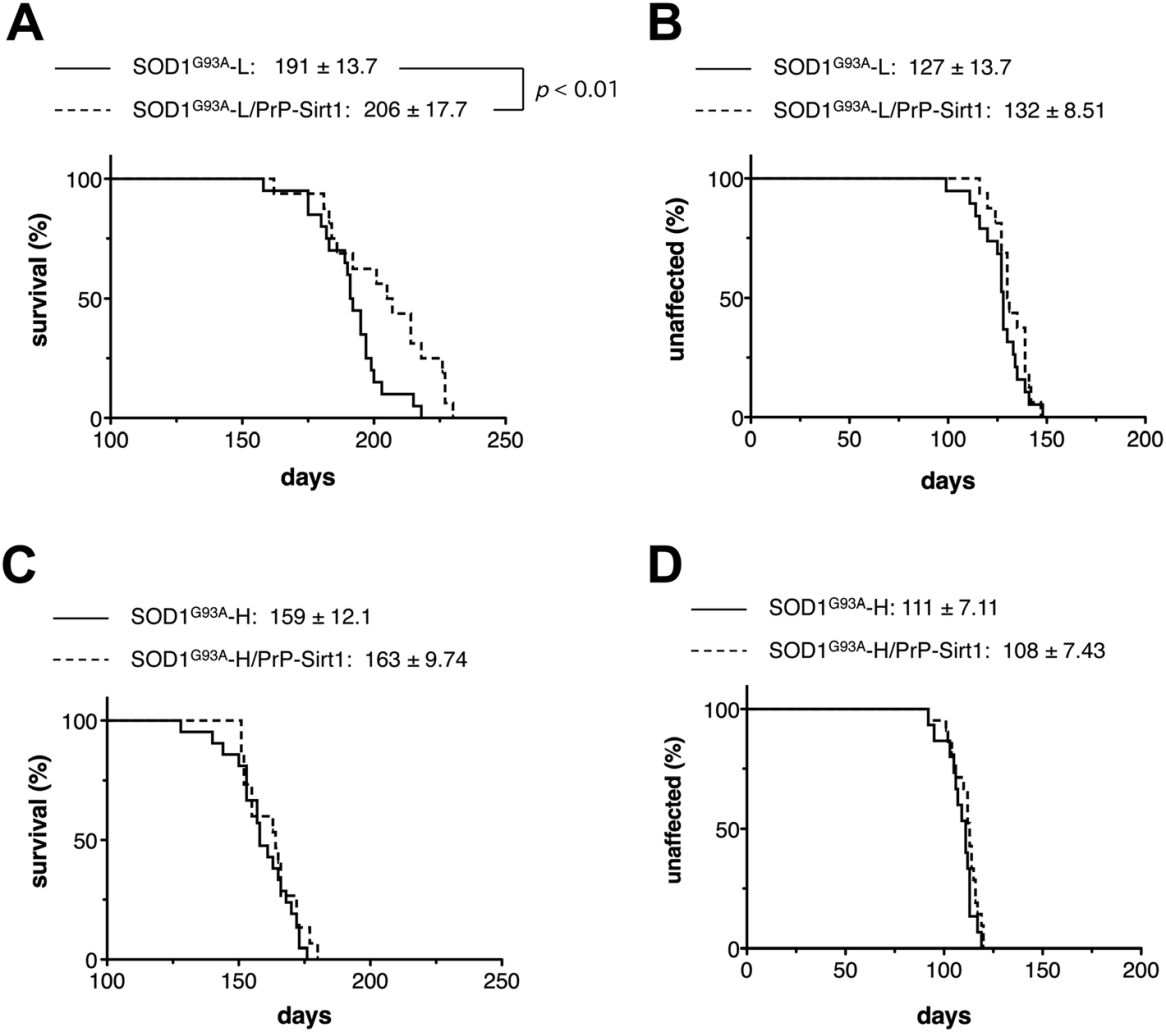
**Effects of SIRT1 overexpression on the lifespan and onset of non-transgenic, and high and low copy SOD1**^**G93A**^**transgenic mice.(AandB)** Survival **(A)** and the onset of the symptom, determined by weight reduction **(B)** of SOD1^G93A^-L (n = 19) and SOD1^G93A^-L/PrP-Sirt1 (n = 16) mouse cohort plotted over time. **(CandD)** Survival **(C)** and the onset of the symptom **(D)** of SOD1^G93A^-H (n = 21) and SOD1^G93A^-H/PrP-Sirt1 (n = 15) mouse cohort plotted over time. The data were analyzed by log-rank test and the average onset and survival time are shown with SD.

**Figure 4:**
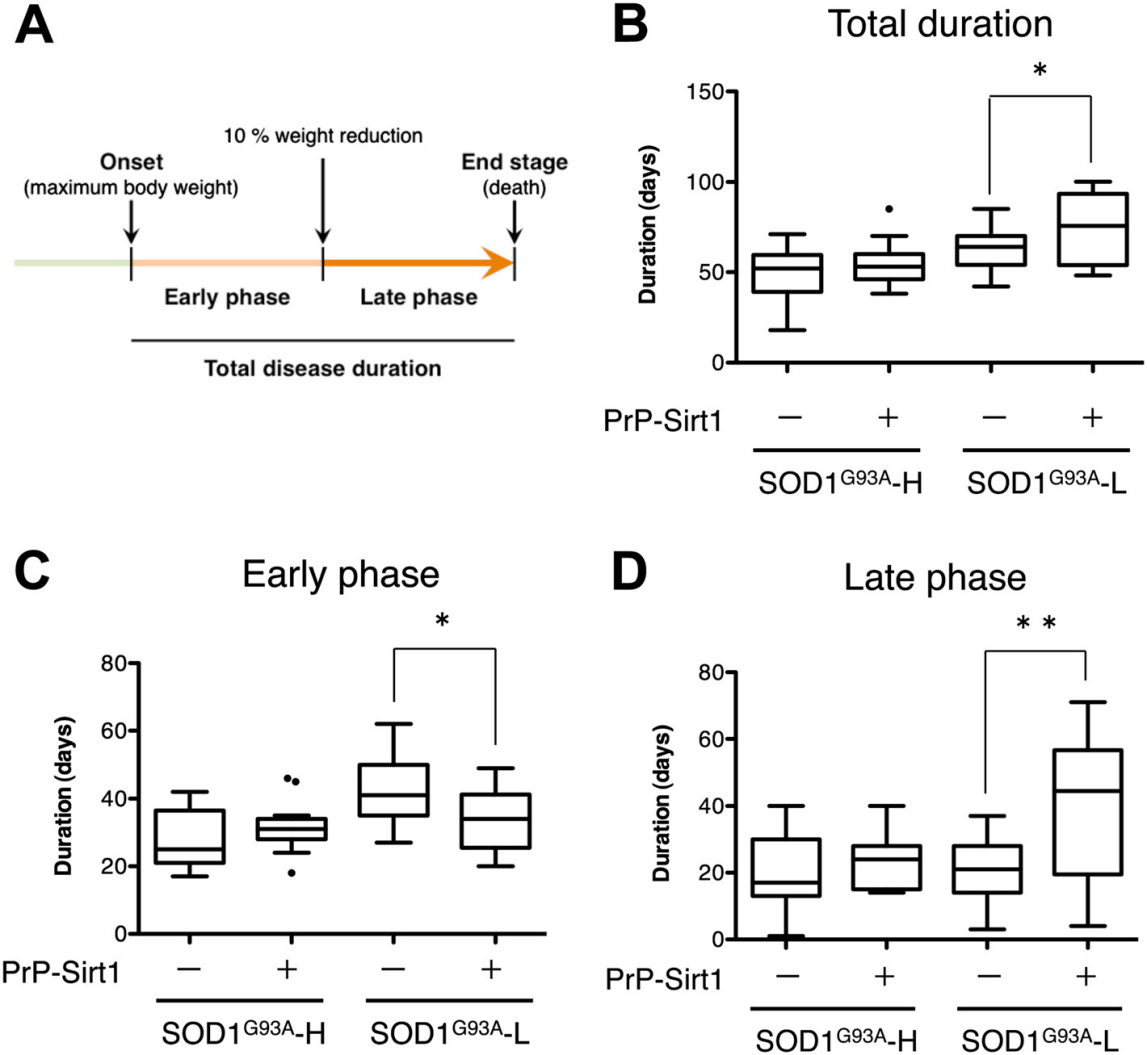
**Effects of SIRT1 overexpression on the disease duration of non-transgenic, and high and low copy SOD1**^**G93A**^**transgenic mice. (A)** Definition of the early and late phases of the disease. **(B-D)** Comparison of the total **(B)**, early **(C)**, and late **(D)** phases of the disease duration. SIRT1 overexpression extended the total disease duration in the SOD1^G93A^-L mice by extending the late phase. The data were analyzed by one-way ANOVA following post-hoc t-tests. *, *p* < 0.05; **, *p* < 0.01.

To examine putative beneficial effects of SIRT1 overexpression on the neurotoxicity by mutant SOD1, we crossbred the PrP-Sirt1 mice with two lines of SOD1^G93A^ transgenic mice; SOD1^G93A^-H and SOD1^G93A^-L with a lower copy number . SOD1^G93A^-L mice express *SOD1*^G93A^ transgene approximately 60% of SOD1^G93A^-H mice (Additional file 1: Figure S2) and survive about 30 days longer on average (Figure 3A). We found that SIRT1 overexpression significantly extended the lifespan of SOD1^G93A^-L mice (Figure 3A) without altering the onset of disease (Figure 3B). In contrast, SIRT1 did not give any beneficial effects on the lifespan (Figure 3C) and the disease onset of SOD1^G93A^-H mice (Figure 3D). The disease progression was analyzed by dividing the total disease duration into the early and late phases (Figure 4A). SIRT1 extended the total disease duration in SOD1^G93A^-L mice (74.6 ± 18.8 days for SOD1^G93A^-L/PrP-Sirt1; 63.2 ± 12.6 days for SOD1^G93A^-L) (Figure 4B). Interestingly, while the early phase duration was slightly reduced (34.3 ± 9.32 days for SOD1^G93A^-L/PrP-Sirt1; 42.0 ± 10.3 days for SOD1^G93A^-L) (Figure 4C), further extension was observed in the late-phase (40.3 ± 20.2 days for SOD1^G93A^-L/PrP-Sirt1; 21.2 ± 8.92 days for SOD1^G93A^-L) (Figure 4D). These data indicate that SIRT1 overexpression was sufficient to slow the disease progression of SOD1^G93A^-L mice, but not of SOD1^G93A^-H mice.

### SIRT1 overexpression causes deacetylation of HSF1, upregulation of HSP70i, and reduction of misfolded SOD1^G93A^

**Figure 5:**
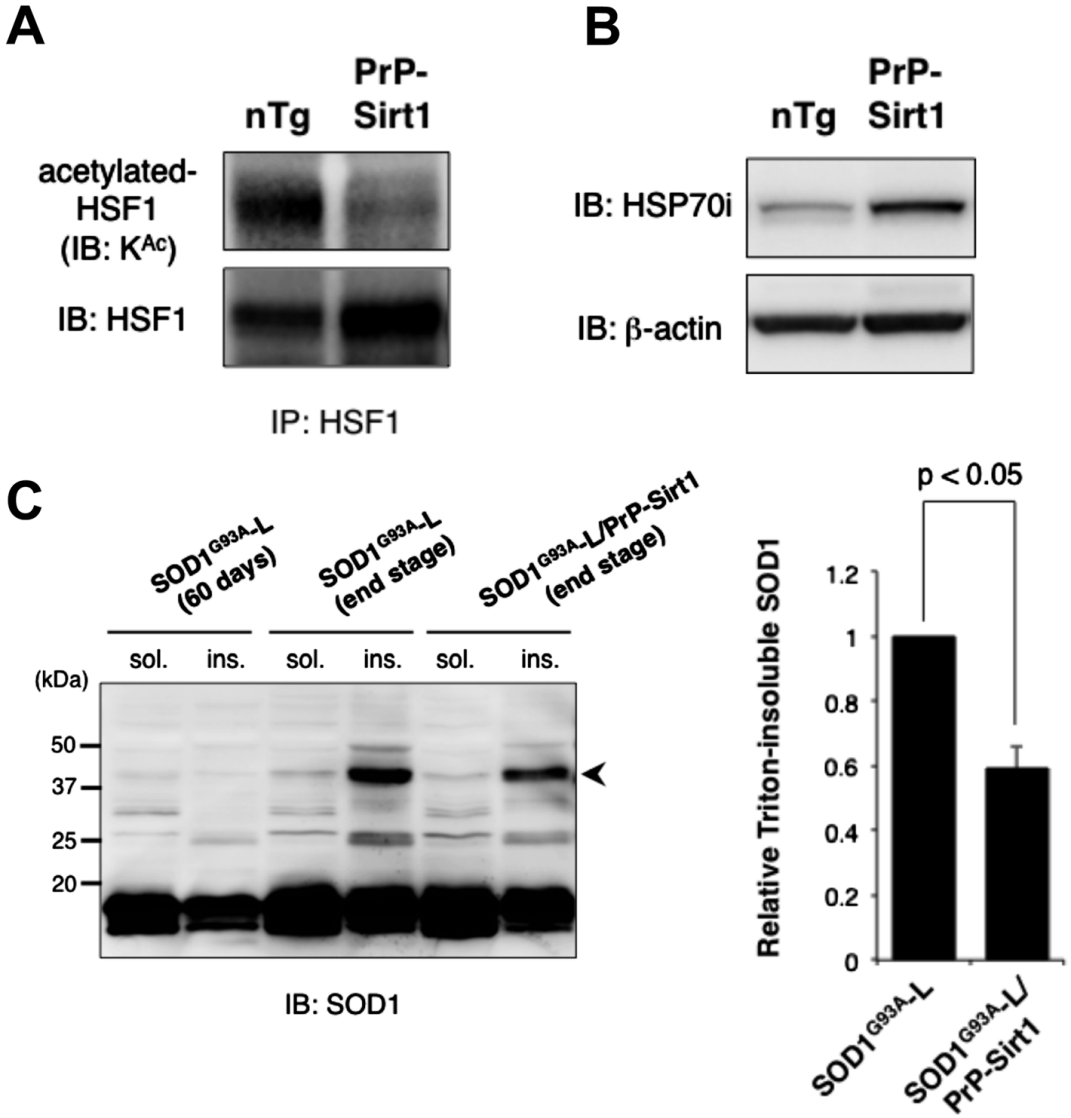
**Effects of SIRT1 overexpression on the induction of HSP70i in the spinal cord of SOD1**^**G93A**^**-L/PrP-Sirt1 transgenic mice. (A)** HSF1 was significantly deacetylated in 5-month-old PrP-Sirt1 mouse spinal cord. Acetylated HSF1 was detected with anti-acetylated lysine antibody after the immunoprecipitation with anti-HSF1 antibody. **(B)** HSP70i was significantly induced in the PrP-Sirt1 mouse spinal cord. **(C)** Sirt1 overexpression reduced the insoluble SOD1 species in the end-stage SOD1^G93A^ mouse spinal cord. Triton X-100 insoluble and SDS-resistant SOD1^G93A^ dimer (arrowhead, ~40 kDa) was detected by immunoblotting (left panel). The relative densitometric values were shown as mean ± SEM (right panel). SIRT1 overexpression significantly suppressed aggregation and insolubilization of SOD1^G93A^. Each lane in **A**, **C**, and **D** contained 15 μg of total protein and the similar results were obtained from three independent experiments.

To assess whether the disease progression is correlated with the deactylation level of HSF1 and/or the expression of HSP70i, we performed immunoprecipitation and immunoblot analysis. In a 5-month-old PrP-Sirt1 mouse spinal cord, the ratio of acetylated HSF1 over total HSF1 was significantly less and the level of HSP70i was significantly higher than those of the littermate (Figure 5A and B). These data suggest direct or indirect activation of the HSF1/HSP70i pathway by SIRT1 overexpression. Despite SIRT1 overexpression, however, HSP70i was not further upregulated in SOD1^G93A^-H, SOD1^G93A^-L mice at the end stage (Additional file 1: Figure S1), implying that HSP70i had been peaked out. To test the putative beneficial effects of SIRT1 overexpression against SOD1^G93A^-mediated proteotoxicity, we measured disease-related dimerization of mutant SOD1. Previous studies showed that mutant SOD1 forms Triton X-100 insoluble and sodium dodecyl sulfate (SDS)-resistant dimer in the spinal cord of transgenic mice at symptomatic phase whose accumulation is inversely correlated with proteasome and chaperone activities [8],[19]. We found that the dimerized SOD1 (approximately 40 kDa) in the spinal cord of a SOD1^G93A^-L/PrP-Sirt1 mouse was less than that of a littermate SOD1^G93A^-L mouse (Figure 5C). Since SDS-resistant SOD1 dimer was not detected in a presymptomatic SOD1^G93A^-L mouse (60 days of age) (Figure 5C, left panel), HSP70i induced through the SIRT1/HSF1 pathway might reduce the accumulation of the toxic SOD1 dimers after the onset of the symptom. These findings suggest that the SIRT1/HSF1/HSP70i pathway contributes to the reduction of toxic misfolded and/or aberrantly dimerized species of SOD1, which accumulate in the late phase of the disease.

## Discussion

In this study, we established a line of transgenic mice that chronically overexpress SIRT1 in the brain and spinal cord. As expected, overexpression of SIRT1 gave partial rescue effect on SOD1^G93A^ mice. Supplementation of SIRT1 in the CNS slowed the disease progression and extended the lifespan of SOD1^G93A^-L mice with a reduction of misfolded/aggregated SOD1 presumably through the activation of the HSF1/HSP70i pathway.

Previous reports from other groups showed that SIRT1 overexpression causes deficits in reference memory in the novel object recognition test [20], voluntary movement in the open field test, and motor coordination in the rotating rod test [21]. In contrast, we detected no significant neurological or behavioral differences between PrP-Sirt1 mice and their non-transgenic littermates with a few minor exceptions (Table 1, Additional file 1: Figure S3-S17). The discrepancy might be due to the transgene constructions, genomic position effects, and epigenetic factors that affect the levels and spatiotemporal patterns of expression. Our PrP-Sirt1 mice with minimal neurological deficits are suitable for testing the effects of SIRT1 supplementation on various models of neurological disorders. In fact, their remarkable resistance against cerebral hypoperfusion has been demonstrated with a bilateral carotid artery stenosis model [22].

The beneficial role of SIRT1 has been demonstrated in various neurodegenerative disease models. Neuroprotective effects of SIRT1 have been reported in Huntington’s disease [23],[24], Parkinson’s disease [25], Alzheimer’s disease [26],[27], and spinal and bulbar muscular atrophy [28]. In this study, we demonstrated that SIRT1 overexpression has partial protective effects on a mouse ALS model. Previous studies showed that resveratrol, an activator of SIRT1, extended the lifespan of SOD1^G93A^ mice [15],[16]. However, given the pleiotropic effects of resveratrol through other target proteins, our results provide the first direct evidence for the contribution of SIRT1 to the alleviation of neurodegeneration in the ALS model.

Our findings suggest that the beneficial effects of SIRT1 depend at least in part on the HSF1/HSP70i pathway. This is consistent with a previous study demonstrating that HSF1 overexpression is partially protective against SOD1-mediated toxicity through the induction of HSP70i and αB-crystallin [29]. On the other hand, it should be noted that this study does not exclude possible involvement of other downstream effectors of SIRT1, such as PGC-1α , or p53, in the neuroprotective effects.

HSP70i reduces Triton X-100-insoluble SOD1 species (Figure 5C) [8] and exogenously delivered HSP70i extends the lifespan of SOD1^G93A^ mice [30]. Although it is unknown whether the mutant SOD1 proteins are refolded, degraded or both in each case, these data suggest the reduction of toxic SOD1 species contributes to the amelioration of SOD1-linked ALS model mice by inducing HSP70i. Since endogenous chaperone system was not sufficiently activated in SOD1-linked ALS model mice (Figure 2C) [9], supplementation of HSPs would be a promising means to cope with the proteotoxicity of mutant SOD1. Another study showed that HSP70i overexpression does not ameliorate the symptom and pathology of SOD1^G93A^, SOD1^G37R^, and SOD1^G85R^ transgenic mouse lines [31]. We speculate that the differences come from the severity of the models. As we demonstrated, SIRT1 overexpression was effective for the rescue of SOD1^G93A^-L but not of SOD1^G93A^-H (Figure 3), whose levels of HSP70i in the end stage were comparable (Additional file 1: Figure S1). These findings suggest that the refolding capacity of HSP70i against the toxicity of mutant SOD1 is limited, especially in the presence of excess amounts of misfolded proteins by overexpression of transgene.

We have previously demonstrated that E6-associated protein (E6-AP), a dual function steroid hormone receptor coactivator and ubiquitin-protein ligase, is involved in SOD1 aggresome degradation and suppresses mutant SOD1-mediated toxicity [32]. Interestingly, E6-AP ubiquitinates the misfolded proteins that are bound to HSC70 [33], and co-expression of E6-AP and HSC70 gives more potent neuroprotection [32]. It is also known that C-terminus of HSP70-interacting protein (CHIP) is involved in misfolded protein degradation through the ubiquitin-proteasome system [34],[35]. These observations suggest that the chaperone system and the ubiquitin-proteasome system are closely coordinated to cope with the proteotoxic stress. Proteasomal function is inhibited by mutant SOD1 [19],[36] and the expression of HSP70i has peaked out at the end stage of the disease even in the case of SOD1^G93A^-L (Additional file 1: Figure S1). These data suggest that accumulation of proteotoxic species caused by proteasomal dysfunction could readily excess the capacity of the chaperone system. Therefore, coordinated activation of the chaperone system and protein degradation systems that include the ubiquitin-proteasome system and the autophagy system is critical to cope with the proteotoxicity of mutant SOD1 or other causative proteins of neurodegenerative diseases.

## Conclusion

The genetic supplementation of SIRT1 can ameliorate a mutant SOD1-linked ALS mouse model partly through the positive regulation of the HSF1/HSP70i chaperone system. Future studies shall include identifying other downstream effectors and testing potential benefits of pharmacological enhancement of the deacetylation activity of SIRT1 after the onset of the symptom.

## Materials and Methods

### Generation of SIRT1 transgenic mice and crossbreeding with mutant SOD1 mice

To generate a line of transgenic mice that chronically overexpress SIRT1 in the central nervous system, we constructed a transcription unit by inserting the coding region of the mouse *Sirt1* cDNA into the MoPrP (mouse prion gene promoter)-polyA cassette (Figure 1A) [37]. We injected linearized MoPrP-*Sirt1* transcription unit with a genetic marker (mouse αA-crystallin promoter-driven enhanced green fluorescent protein (EGFP)) into C57BL/6 J mouse oocytes and obtained a founder that transmitted the transgene in Mendelian manner. The genotyping was done by polymerase chain reaction for an artificial sequence near the MoPrP-*Sirt1* junction. The transgenic line is to be deposited to RIKEN Bioresource Center. Sirt1 transgenic and non-transgenic control mice for behavioral analyses were bred as littermates. Transgenic mouse lines expressing human SOD1 gene with ALS-linked mutations, SOD1^G93A^-H [B6.Cg-Tg(SOD1*G93A)1Gur/J] or loxSOD1^G37R^ [B6.Cg-Tg(SOD1-G37R) 1Dwc/J] were previously described [17],[18]. SOD1^G93A^-L was generated by a spontaneous drop of the copy number of the SOD1^G93A^-H line, and has been maintained in our laboratory for several generations.

### Animals and experimental design

All animal procedures were conducted in accordance with the guidelines of the Animal Use and Care Committees of Kyoto University, National Institute for Physiological Science, Nagoya University, and RIKEN. All comparisons were made between littermates to minimize confounding effects of different genetic background and environment. All behavioral tests were conducted at 5–9 months of age as described previously [38],[39]. Mice were group housed (4 mice per cage) in a specific pathogen-free room kept at 23 ± 2°C with a 12 h light/dark cycle (lights on at 7 a.m.) with access to food and water. For survival experiments, SOD1^G93A^ mice were always compared with their littermates with PrP-Sirt1 gene. Times of disease onset, early phase, and end stage were determined respectively as the time when mice had started losing the maximum body weight, when denervation-induced muscle atrophy had produced a 10% loss of maximal weight, and when animals in lateral position failed in righting within 20 seconds, an endpoint commonly used for mutant SOD1 expressing mice which is compliant with the requirements of the Animal Use and Care Committee. Disease progression was defined by the duration between the onset and disease end-stage. Statistical analysis of survival time and disease duration was performed with a log-rank test and one-way ANOVA following post-hoc t-tests, respectively. Analyses were carried out by using GraphPad Prism (GraphPad Software, La Jolla, CA).

### Genotyping of mice

Mice harboring *PrP-Sirt1* transgene were identified by PCR using following primers; 5′-CAAGAGGTTGTTAATGAAGC-3′ and 5′-TTTCCTGTTGCCTTCAATCAGCTATCG-3′.

Mice harboring *SOD1*^G93A^ or *SOD1*^G37R^ transgene were identified by PCR using following primers; the mouse *SOD1* gene fragment (850 bp) was amplified by mSOD-A primer: 5′-GTTACATATAGGGGTTTACTTCATAATCTG-3′ and h/mSOD-C primer: 5′-CAGCAGTCACATTGCCCARGTCTCCAACATG-3′, and human SOD1 gene fragment (750 bp) was amplified by hSOD-B primer: 5′-CCAAGATGCTTAACTCTTGTAATCAATGGC-3′ and h/mSOD-C primer.

### Antibodies

We used the following commercially available antibodies: anti-SIRT1 from EMD Millipore Corp. (Billerica, MA, USA), anti-β-actin from Sigma-Aldrich Co. LLC. (St. Louis, MO, USA), anti-acetylated lysine from Cell Signaling Technology Inc. (Danvers, MA, USA), anti-HSF1, anti-HSP70i and anti-HSP90 from Enzo Life Sciences Inc. (Farmingdale, NY, USA). Rabbit anti-human SOD1 antibody was raised against a recombinant human SOD1 peptide (24–36) and purified with protein A [40].

### Immunoblotting

Tissues were homogenized in TNE lysis buffer [50 mM Tris–HCl (pH 7.4), 150 mM NaCl, 1 mM ethylenediaminetetraacetic acid (EDTA), 1% Triton-X 100, protease inhibitor cocktail (Roche, Basel, Switzerland)] with Dounce homogenizer. The lysates were centrifuged at 15,000 × g for 10 min at 4°C and the supernatants were collected. For the analyses of insoluble SOD1, the pellets were re-solubilized in the equivalent volume of TNE lysis buffer supplemented with 2% SDS and benzonase (Roche). After minutes of incubation at room temperature, the lysates were sonicated and centrifuged at 15,000 × g for 10 min. The supernatants were collected as insoluble fractions. All the protein content was measured by micro BCA assay kit (Thermo Fisher Scientific Inc., Waltham, MA, USA) and equal amounts of total proteins were analyzed by immunoblotting. Densitometric analysis was performed after the chemiluminescence detection by using an image analyzer LAS-4000mini (Fuji film, Tokyo, Japan) with the equipped software.

### Immunoprecipitation

For the detection of HSF1 deacetylation, we slightly modified the previously reported procedure [14]. In brief, lumbar spinal cords were homogenized in radioimmunoprecipitation (RIPA) buffer [50 mM Tris–HCl (pH 8.0), 150 mM NaCl, 1% NP-40, 0.5% sodium deoxycholate, 0.1% SDS] supplemented with 1 mM EDTA, 2 mM nicotinamide and 1 mM trichostatin A (all from Sigma) with Dounce homogenizer. The lysates were centrifuged at 15,000 × g for 10 min at 4°C. Anti-HSF1 antibody (1:100) was added to the supernatant and incubated for over-night at 4°C with gentle agitation. Protein G sepharose (GE Healthcare, Waukesha, WI, USA; 15 μl each) was added and incubated for further 1.5 h. The beads were washed four times with phosphate buffered saline. The proteins were eluted with 2 × SDS loading buffer and heating at 95°C for 3 min. Deacetylated and total HSF1 were detected on the same polyvinylidene difluoride membrane with Restore western blot stripping buffer (Thermo).

## Abbreviations

ALS: amyotrophic lateral sclerosis

CNS: central nervous system

SOD1: Cu/Zn-superoxide dismutase

EGFP: enhanced green fluorescent protein

HSF1: heat shock factor 1

HSP: heat shock protein

HSP70i: inducible heat shock protein 70

SD: standard deviation

SEM: standard error of the mean

SOD1^G93A^-H: SOD1^G93A^ high expression line

SOD1^G93A^-L: SOD1^G93A^ low expression line

SDS: sodium dodecyl sulfate

## Competing interests

The authors declare that they have no competing interests.

## Authors’ contributions

SW, SN, and NA-I conducted the biochemical, histological, behavioral analyses with supports from OK, FE, HM, and RT, under the supervision of KY and MK. KT and TM supervised the behavioral tests. MK and KY designed this study. SW, MK and KY wrote the manuscript. All authors read and approved the manuscript.

## Additional file

## Supplementary Material

Additional file 1 Additional experimental procedures and figures. Click here for file
