# Supplementary material for: SIRT1 overexpression ameliorates a mouse model of SOD1-linked amyotrophic lateral sclerosis via HSF1/HSP70i chaperone system
Source: Mol Brain. 2014 Aug 29;7:62. doi: 10.1186/s13041-014-0062-1 (PMC4237944; doi:10.1186/s13041-014-0062-1)

Additional File 1

SIRT1 overexpression ameliorates a mouse model of SOD1-linked amyotrophic lateral sclerosis via HSF1/HSP70i chaperone system

Seiji Watanabe, Natsumi Ageta-Ishihara, Shinji Nagatsu, Keizo Takao, Okiru Komine, Fumito Endo, Tsuyoshi Miyakawa, Hidemi Misawa, Ryosuke Takahashi, Makoto Kinoshita, and Koji Yamanaka

1. Additional Experimental Procedures

Systematic Behavioral Analysis

We applied our standard protocols [1] to a cohort of male non-transgenic (nTg) andPrP-Sirt1 transgenic (PrP-Sirt1) littermates (n=19, 21) reared in the same cages: Behavioral testing was conducted between 9 a.m. and 6 p.m. except for the continuous home cage monitoring. Each apparatus was cleaned with sodium hypochlorite solution to minimize odor after use. We conducted tests in the following order; general health and neurological screening (including body weight and temperature measurements, grip strength test, righting test, whisker touch test, and ear twitch reflexes, wire hang test), light/dark transition test, open field test, elevated plus maze test, hot plate test, one-chamber social interaction test, rotarod test, acoustic startle response/prepulse inhibition test, Porsolt forced swim test, Barnes maze test, three-chamber sociability and preference for social novelty test, fear conditioning test, gait analysis, tail suspension test, and long-term monitoring of locomotion and social interaction in home cage. Intervals between tests were > 24 h.

Neuromuscular strength tests

Neuromuscular strength was assessed with the forelimb grip strength test and wire hang test. Forelimb grip strength was measured by pulling a mouse in the tail while its forepaws hung on to a wire grid attached to a spring balance. The tensile force (N) when the mouse released the grid was measured three times, and the greatest value was analyzed. In the wire hang test, a wire mesh with a mouse on top was slowly inverted and the latency to fall was measured.

Light/dark transition test

The apparatus had a pair of differentially illuminated (390 lux vs. 2 lux) chambers (21 × 41 × 25 cm) connected with a door in the middle. Each mouse was released in the dark chamber, and image data were acquired from the top with a CCD camera for 10 min. The latency until the first entry into the light chamber, the time spent in each chamber, the number of transitions, and the total distance traveled were automatically measured using ImageLD software (see Image analysis).

Open field test

Voluntary locomotor activity was measured in an open field test. Each mouse was placed in the center of the open field apparatus (40 x 40 x 30 cm; Accuscan Instruments) illuminated at 100 lux. The following indices were monitored for 120 min; total distance traveled, time spent in the center area of 20 × 20 cm, number of rearing and beam-breaks were automatically measured by counting interruptions of infrared beams.

Elevated plus maze test

The apparatus had two open arms (25 x 5 cm, with 3-mm-high plastic ledges) and two closed arms (25 x 5 cm, with 15-cm-high transparent walls) interconnected via a central crossing (5 x 5 cm), which was set at 55 cm-height and illuminated at 100 lux. The numbers of entries into, and the time spent in the open and enclosed arms, were recorded for 10 min. Image data were acquired from the top with a CCD camera, and the number of entries into and the time spent in the open/closed arms, and total distance traveled were measured automatically using ImageEP software (see Image analysis).

Acoustic startle response and PPI test

A mouse restrained in a cylinder was placed in the chamber of a startle reflex measurement system (O'Hara & Co.) with 70 dB background white noise. After 10 min, the mouse's startle response to a startle stimulus (110 or 120 dB white noise for 40 ms) was measured by a motion sensor for 140 ms. A test session was a random sequence of four trials each with a prepulse stimulus (74 or 78 dB white noise for 40 ms that preceded the startle stimulus by 100 ms) and two without. Six blocks of 6 trials were presented in pseudorandom order with the average inter-trial interval of 15 s.

Porsolt forced swim test

Each mouse was released in 7.5-cm-deep water at 23˚C in an acrylic cylinder (10 cm in diameter), and the duration of the motion for evacuation was measured up to 10 min automatically using ImagePS software (see Image analysis).

Monitoring social interaction and voluntary activity in the home cage

The position of each mouse housed alone in a cage was monitored from the top continuously for a week. The distance traveled along the diurnal cycle was measured automatically using ImageHA software (see Image analysis). Two mice of the same genotype that had been separately reared were housed together in a home cage and their 2D images from the top were captured at 1 fps for a week. Their physical contact and separation were represented respectively as 1 and 2 particles, and their locomotor activity was quantified by the differentials of pixels between successive frames by using ImageHA software (see Image analysis).

One-chamber social interaction test

The positions of two mice placed in a novel chamber (40 x 40 x 30 cm) were monitored from the top at 1 frame/sec. Their horizontal distance traveled and the number of contacts were measured automatically using ImageSI software (see Image analysis).

Crawley's three-chamber test for sociability and preference for social novelty

The apparatus had three chambers (20 × 40 × 22 cm) separated by two transparent partitions each with an opening (5 × 3 cm), and a lid with an infrared CCD camera. A male mouse (5–9 weeks old C57BL/6J, termed Stranger 1) that had no prior contact with the subject mice was enclosed in a cylinder cage (9 cm in diameter, set in the left chamber) that allowed nose contacts. Each subject mouse was released in the middle chamber and allowed to explore for 10 min, while the time spent in each chamber and within 5 cm from each cage was measured automatically using ImageCSI software (see Image analysis). Subsequently, another unfamiliar mouse (Stranger 2) was placed in another cylinder cage (in the right chamber) and monitored likewise for another 10 min.

Rota-rod test

Motor coordination and motor leaning were tested by measuring the survival duration on a 3-cm-thick rotating rod which was accelerated from 4 to 40 rpm over 5 min. Each mouse was subjected to 6 trials over 2 days.

Gait analysis

Automated gait analysis was conducted with DigiGait (Mouse Specifics Inc.). Each mouse was forced to walk on a transparent treadmill moving at 24 cm/sec, when the mouse body movement and paw footprints were captured at 150 frames/sec from underneath the treadmill. Multiple quantitative parameters (length, width and timing of the strides, paw angle etc.) were extracted from the time-lapse images with bundled software.

Hot plate test

Sensitivity and responses to a noxious stimulus were assessed by measuring the latency to the first response after placing a mouse on a metal plate at 55°C.

Tail suspension test

The movement of each mouse suspended by the tail at a height of 30 cm was recorded for 10 min and analyzed by using ImageTS software (see Image analysis).

Contextual and cued fear conditioning tests

Each mouse was exposed to a test chamber (26 x 34 x 33 cm) for 2 min, then to three pairs of a cue (55 dB white noise for 30 sec) each followed by a mild footshock (0.3 mA for 2 sec), repeated at 2-min intervals. For the context testing after 1 or 8 days, freezing was measured in the same chamber. For the cued testing in a distinct spatial context after 1 or 8 days, freezing after the noise was measured in a triangular chamber (35 x 35 x 41 cm) in a different room. The control of the stimuli, image acquisition at 1 frame/sec from the top, and image analysis were done automatically with ImageFZ software (see Image analysis). The criterion of freezing was defined when the difference of binarized mouse areas from two consecutive frames was below 10 pixels and lasted for 2 sec or longer.

Image analysis

The application programs for behavioral data acquisition and analysis (ImageLD, EP, CSI, PS, FZ, TS, HA) were created on the platform of NIH Image (http://rsb.info.nih.gov/nih-image/) and ImageJ (http://rsb.info.nih.gov/ij/) by TM. ImageLD, EP, and FZ are freely available from http://www.mouse-phenotype.org/software.html.

Statistical analysis for behavioral tests

Quantitative data were expressed as mean ± SEM, and either one-way ANOVA or two-way repeated measures ANOVA was applied for statistical analyses unless otherwise noted.

Quantitative PCR

Copy numbers of human *SOD1*^{G93A} transgene in the genomic DNA extracted from the mouse tails were quantified with human *SOD1* specific primers (5’-CAATGTGACTGCTGACAAAG-3’ and 5’-GTGCGGCCAATGATGCAAT-3’) and *β-actin* primers (5’-TTGGCCTCACTGTCCACCTT-3’ and 5’-CGGACTCATCGTACTCCTGCTT-3’). The transgene copy numbers normalized with the *β-actin* gene dosage were expressed as relative values against SOD1^{G93A}-H line.

2. Additional Figure Legends

Additional Figure A1. Expression of HSP70i and SIRT1 in SOD1^{G93A}/SIRT1 double transgenic mouse spinal cord and brain

A representative immunoblot image for HSP70i and SIRT1 in the tissues of SOD1^{G93A}-L, -H and/or PrP-Sirt1 mouse at the end-stage of the disease. Exogenous SIRT1 proteins derived from transgene were clearly detected in the brain and lumbar spinal cord. Each lane contained 20 μg of total protein. Similar results were obtained from three independent experiments.

Additional Figure A2. Copy numbers of SOD1^{G93A} transgene in SOD1^{G93A}-L and -H lines

The relative copy numbers of SOD1^{G93A} transgene in the lumber spinal cord of SOD1^{G93A}-L (n=8), -H (n=4) and non-transgenic (n=2) mice were analyzed by quantitative PCR. In order to confirm the inherited SOD1^{G93A} copy number reduction in the SOD1^{G93A}-L line, the parents (n=2) were also analyzed.

Additional Figure A3-A17. Systematic physical and behavioral analysis of PrP-Sirt1 mice:

Additional Figure A3. Body weight, rectal temperature, and the muscle strength tests

(A) Body weight, (B) rectal temperature, (C) grip strength, and (D) wire hang latency of nTg(wild type) and PrP-Sirt1 mice (n=19, 21). The lighter body weight of PrP-Sirt1 mice

(p=0.023) is reflected in their significantly longer hanging duration (p=0.028).

Additional Figure A4. The light/dark transition test

(A) Distance traveled in the light and dark chambers, (B) latency until the first entry into the light chamber, (C) time spent in the light chamber, and (D) number of transitions across the light/dark border of nTg and PrP-Sirt1 mice (n=19, 21). PrP-Sirt1 mice moved between chambers significantly less frequently than (p=0.042), the reason of which is currently unknown.

Additional Figure A5. The open field test

(A) The total distance traveled in the first 120 min after entry into a novel light chamber. The exploratory locomotive activity of PrP-Sirt1 mice consistently exceeded that of nTg mice (p=0.049, n=21, 19). (B-D) Albeit below the level of statistical significance, hyperactive trend of PrP-Sirt1 mice was also indicated by the longer time spent near the center of the chamber (B), and the increased frequency of rearing events (C) and stereotypic movements (D). (See also Additional Figure A7.)

Additional Figure A6. The elevated plus maze test

(A) Distance traveled, (B) total number of entries into open and closed arms, (C) percentage of entries into open arms, and (D) percentage of stay time on open arms of nTg and PrP-Sirt1 mice (n=19, 21). No statistically significant difference was observed.

Additional Figure A7. The acoustic startle test and prepulse inhibition test

(A) Startle amplitude (arbitrary unit) against acoustic stimuli of two distinct loudness (110 dB or 120 dB), and (B) percent reduction of startle amplitude in the presence of a preceding acoustic stimulus (prepulse of 110 dB or 120 dB) of nTg and PrP-Sirt1 mice (n=19, 21). No statistically significant difference was observed.

Additional Figure A8. Porsolt forced swim test

(Top) Percent immobility, and (bottom) distance traveled of nTg and PrP-Sirt1 mice (n=19, 21) floating in water. No statistically significant difference was observed.

Additional Figure A9. Diurnal social interaction and locomotor activity monitoring in the home cage

Diurnal oscillation of the social interaction (top) and locomotor activity (bottom) of nTg and PrP-Sirt1 mice (n=6, 8 pairs) in the home cage through the light/dark phases. The hyperactive trend at the activity peaks is concordant with the observations in the open field test (Additional Figure A5).

Additional Figure A10. The social interaction test (single chamber)

(A) Schematic diagrams of the single-chamber social interaction test. (B) Distance traveled, (C) total number of contacts, (D) total duration of contacts, (E) total duration of active contacts, of nTg and PrP-Sirt1 mice (n=9, 10 pairs). No statistically significant difference was observed.

Additional Figure A11. The social interaction test (three chambers)

(A) Schematic diagrams of the three-chamber sociability and social novelty preference tests. (Left) The sociability test setup. Each mouse was scored for the time spent in the middle habituated chamber (M), the left chamber containing an unfamiliar C57BL/6J mouse (Stranger 1, S1) in a wire cage, or the right chamber with an empty wire cage (E). (Right) The social novelty preference test following the sociability test uses the same apparatus, except for a novel unfamiliar C57BL/6J mouse (Stranger 2, S2) caged in the right chamber in addition to the now-familiar C57BL/6J mouse (Stranger 1, S1) remaining in the left cage/chamber. (B, C) There was no difference in the sociability indices (S1 over E) between nTg and PrP-Sirt1 mice (n=19, 21). (D, E) In the social novelty preference test (Step2) after the sociability test (Step1), PrP-Sirt1 mice exhibited significantly less preference for S2 over S1 than nTg mice (p=0.038), indicative of their reduced curiosity in the novel social stimuli and/or augmented persistence to

the previously-exposed social stimuli.

Additional Figure A12. The rotating rod (Rota-rod) test

The duration in which nTg and PrP-Sirt1 mice (n=19, 21) kept pace with a rotating rod with a constant acceleration increased during 6 trials in two days. No statistically significant difference was observed in the motor coordination and motor learning.

Additional Figure A13. The gait analysis

(A-I) Gait parameters extracted from high-speed digital images of nTg and PrP-Sirt1 mice on a treadmill (n=19, 21). No statistically significant difference was observed except for the wider hind step angle of PrP-Sirt1 mice (G).

Additional Figure A14. The hot plate test

Avoidance responses of nTg and PrP-Sirt1 mice (n=19, 21) to heat (55°C) given to the paws. No statistically significant difference was observed.

Additional Figure A15. The tail suspension test

Immobility time of nTg and PrP-Sirt1 mice (n=19, 21) suspended in the tail. No statistically significant difference was observed.

Additional Figure A16. Barnes maze test

(A, B) Time spent near the correct escape hole (target) was measured at 24 h (A) and 1 month (B) after the training session. No statistically significant difference in the acquisition and retention of spatial memory was observed between nTg and PrP-Sirt1 mice (n=19, 21).

Additional Figure A17. The contextual and cued fear conditioning test

(A) Freezing during the acquisition of the association between a 2-second electric shock in the paws and a preceding tone (cue) in chamber A (context). (B and D) Freezing after being housed in chamber A without the tone 1 or 8 days after the conditioning. (C and E) Freezing after the

tone in chamber B 1 or 8 days after the conditioning. No statistically significant difference was observed between nTg and PrP-Sirt1 mice (n=19, 21).

Reference

1. Ageta-Ishihara N, Yamakado H, Morita T, Hattori S, Takao K, Miyakawa T, Takahashi R, Kinoshita M: **Chronic overload of SEPT4, a parkin substrate that aggregates in Parkinson's disease, causes behavioral alterations but not neurodegeneration in mice.** *Mol Brain* 2013, **6:** 35.

Additional Figure A1

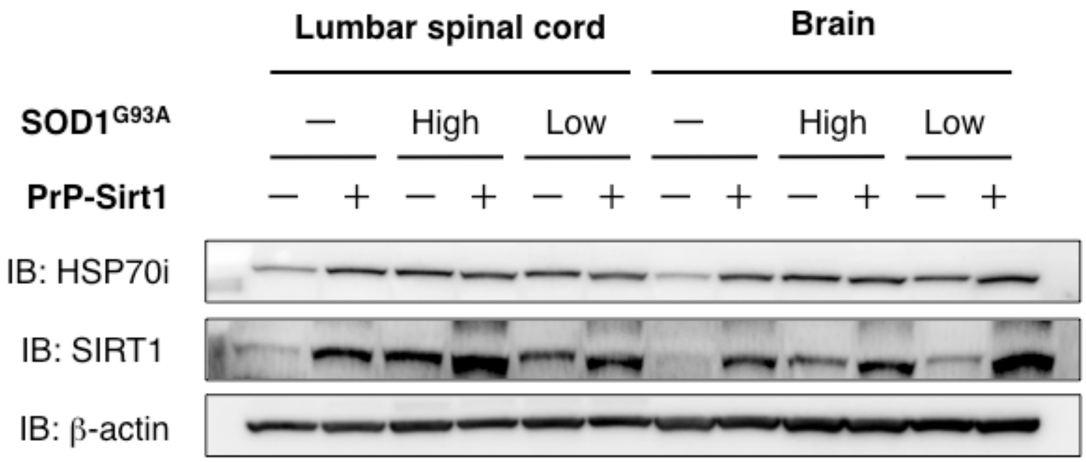

Additional Figure A2

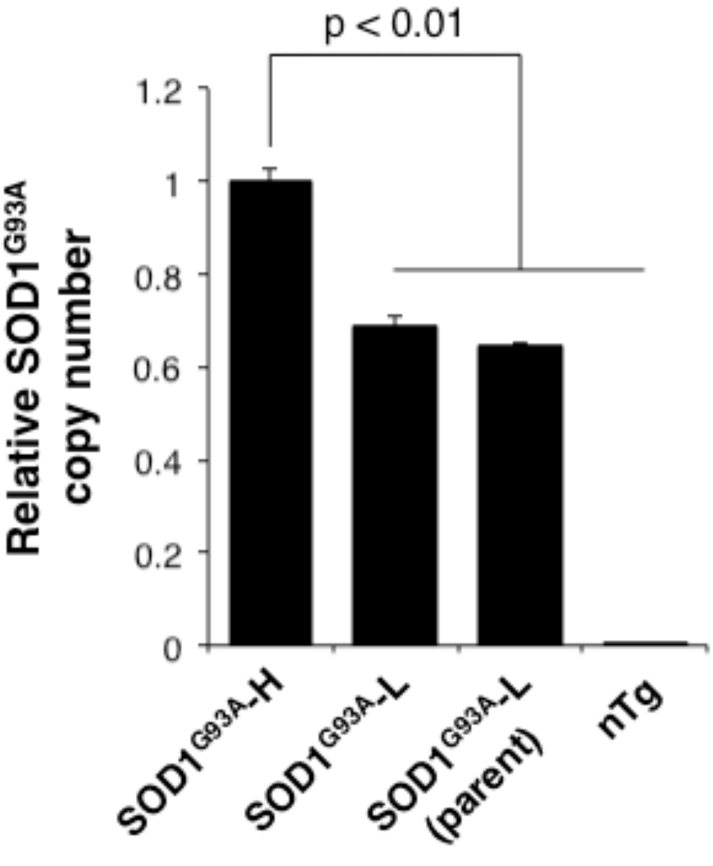

Additional Figure A3

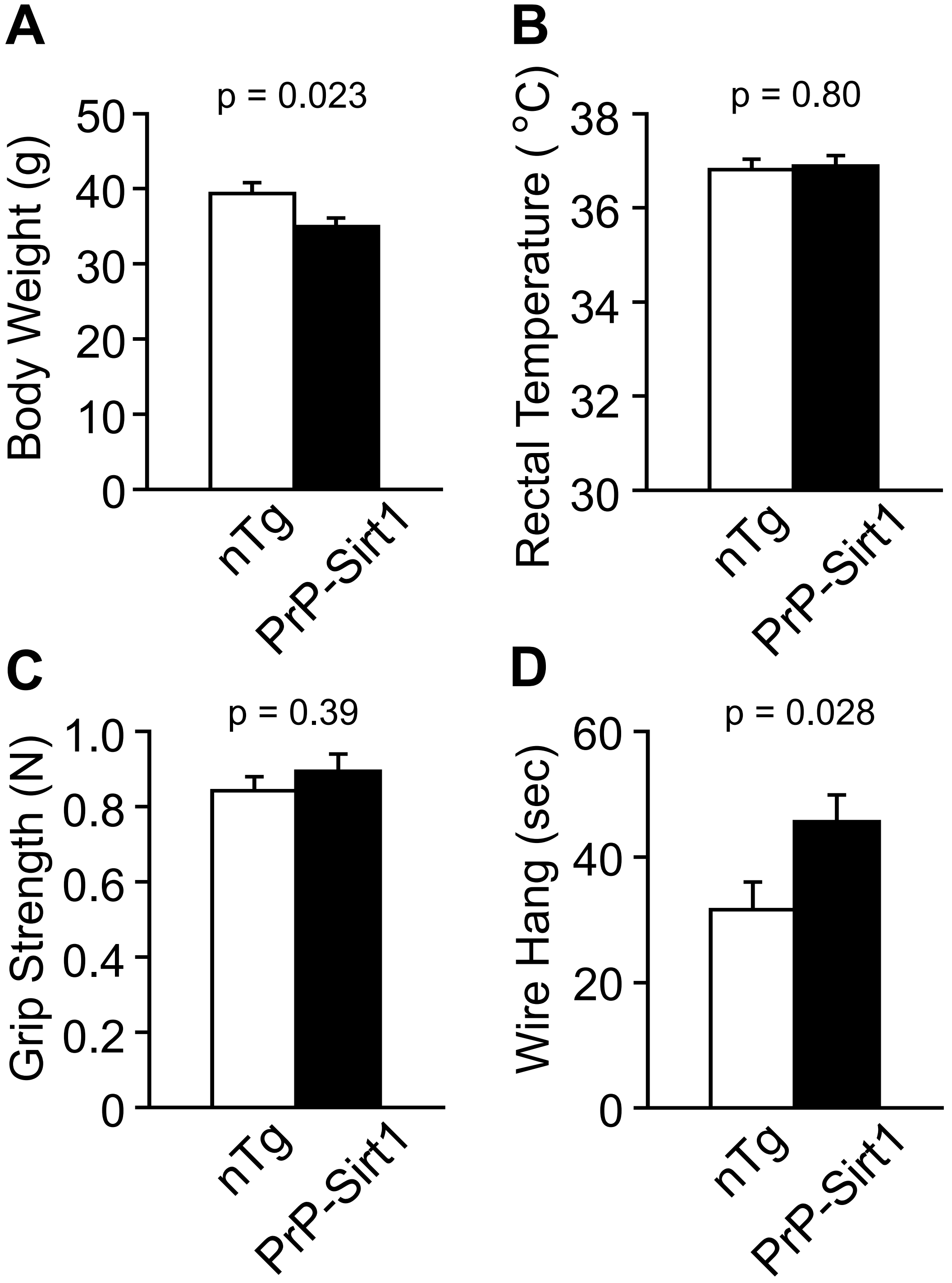

Additional Figure A4

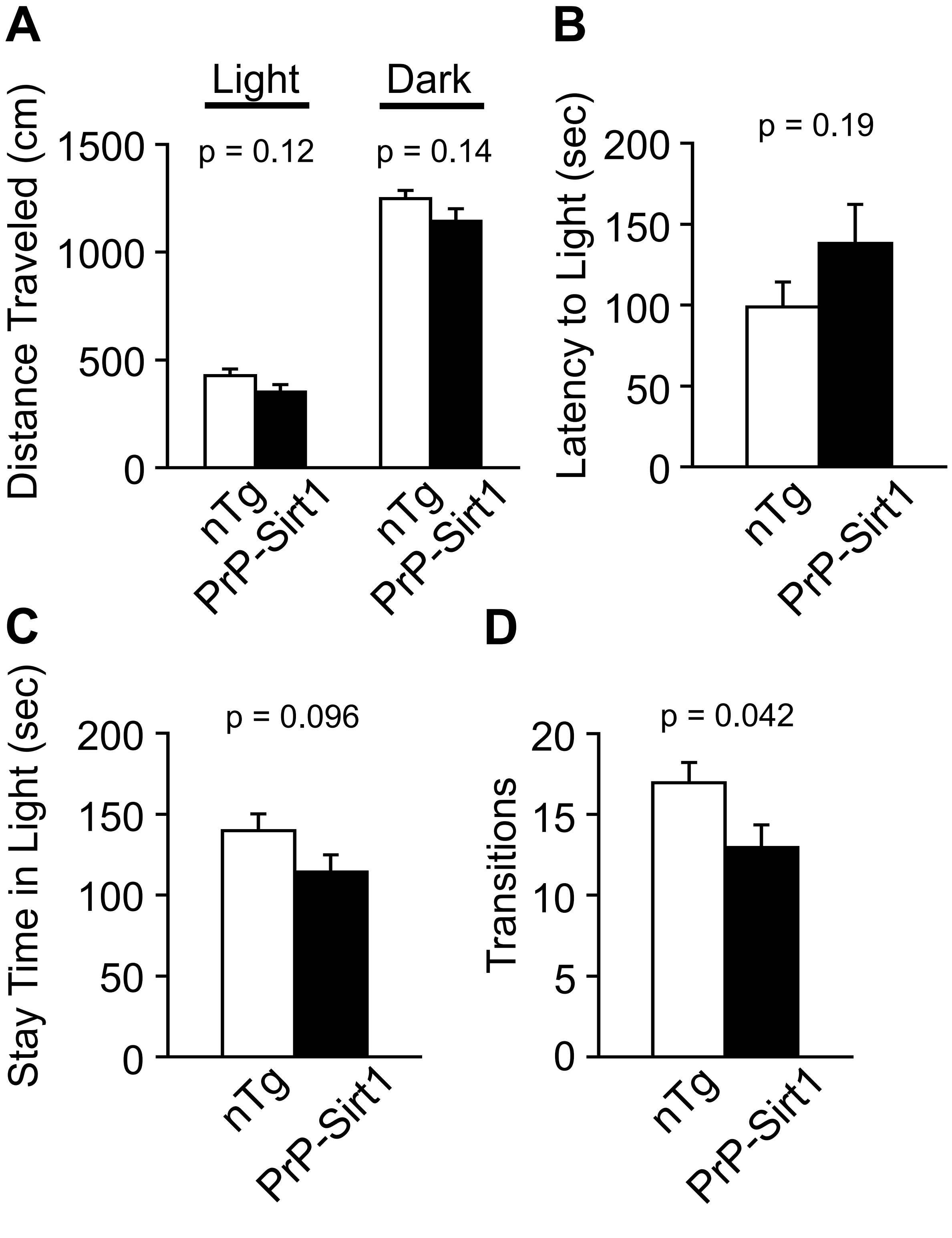

Additional Figure A5

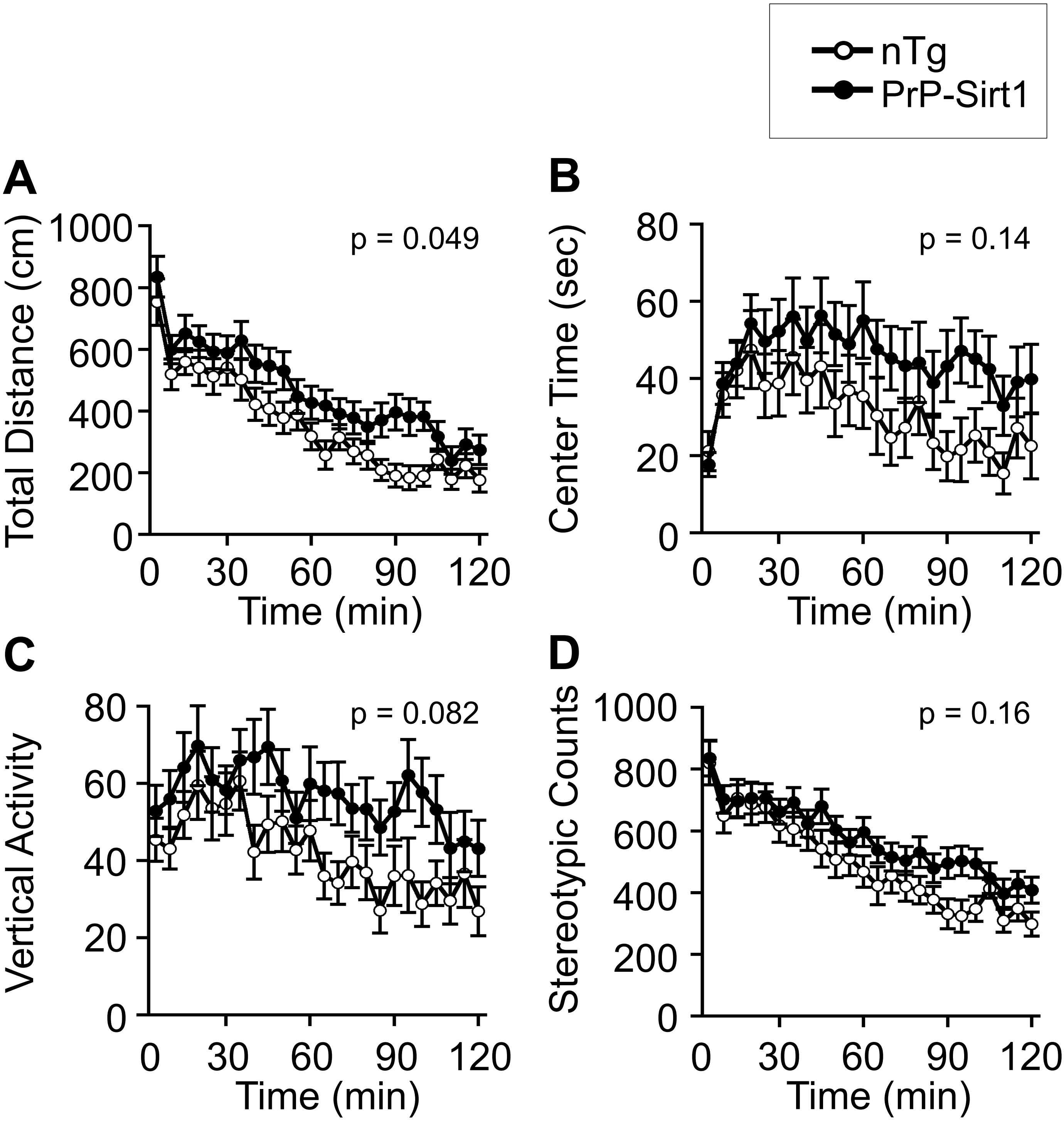

Additional Figure A6

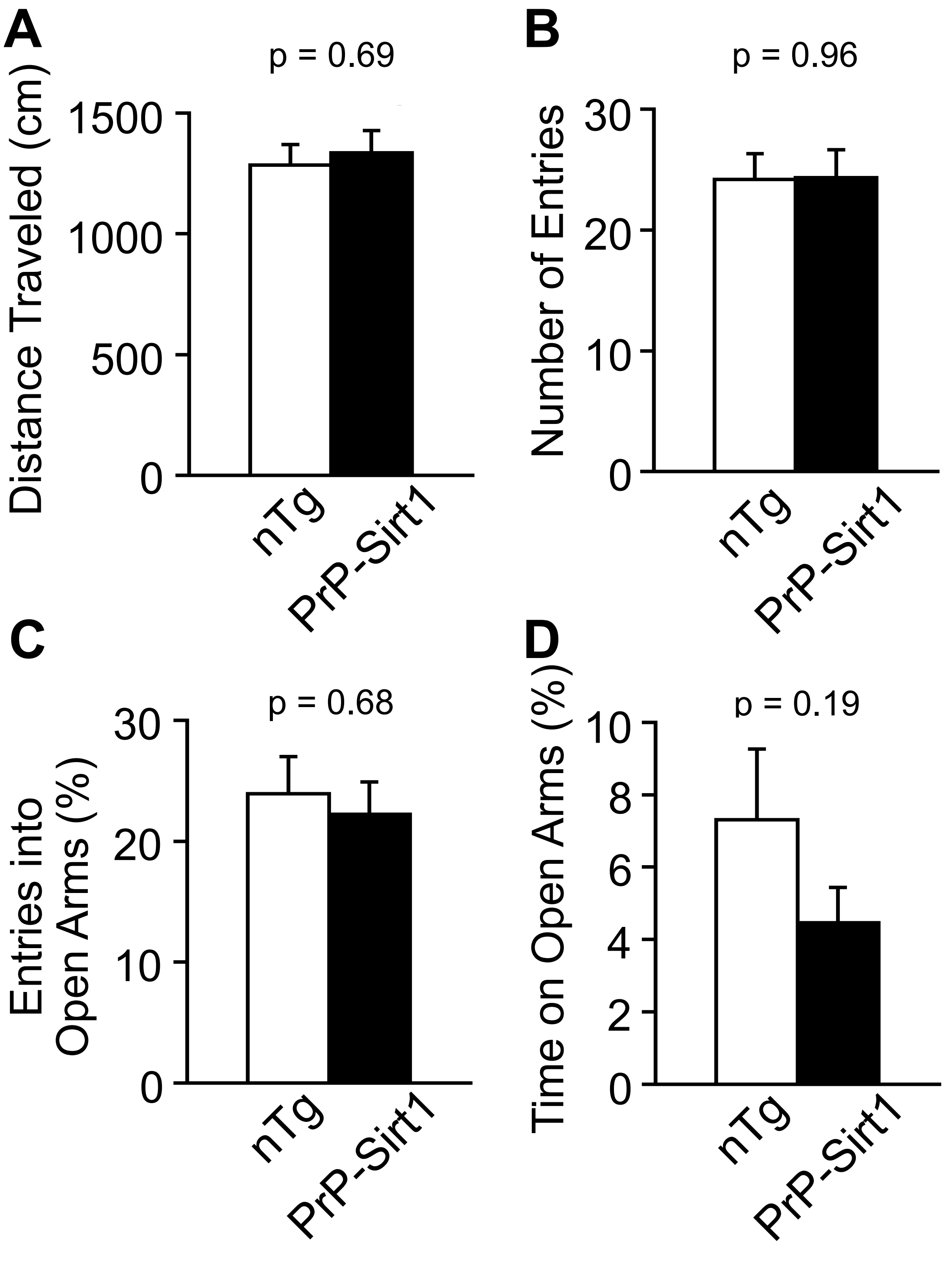

Additional Figure A7

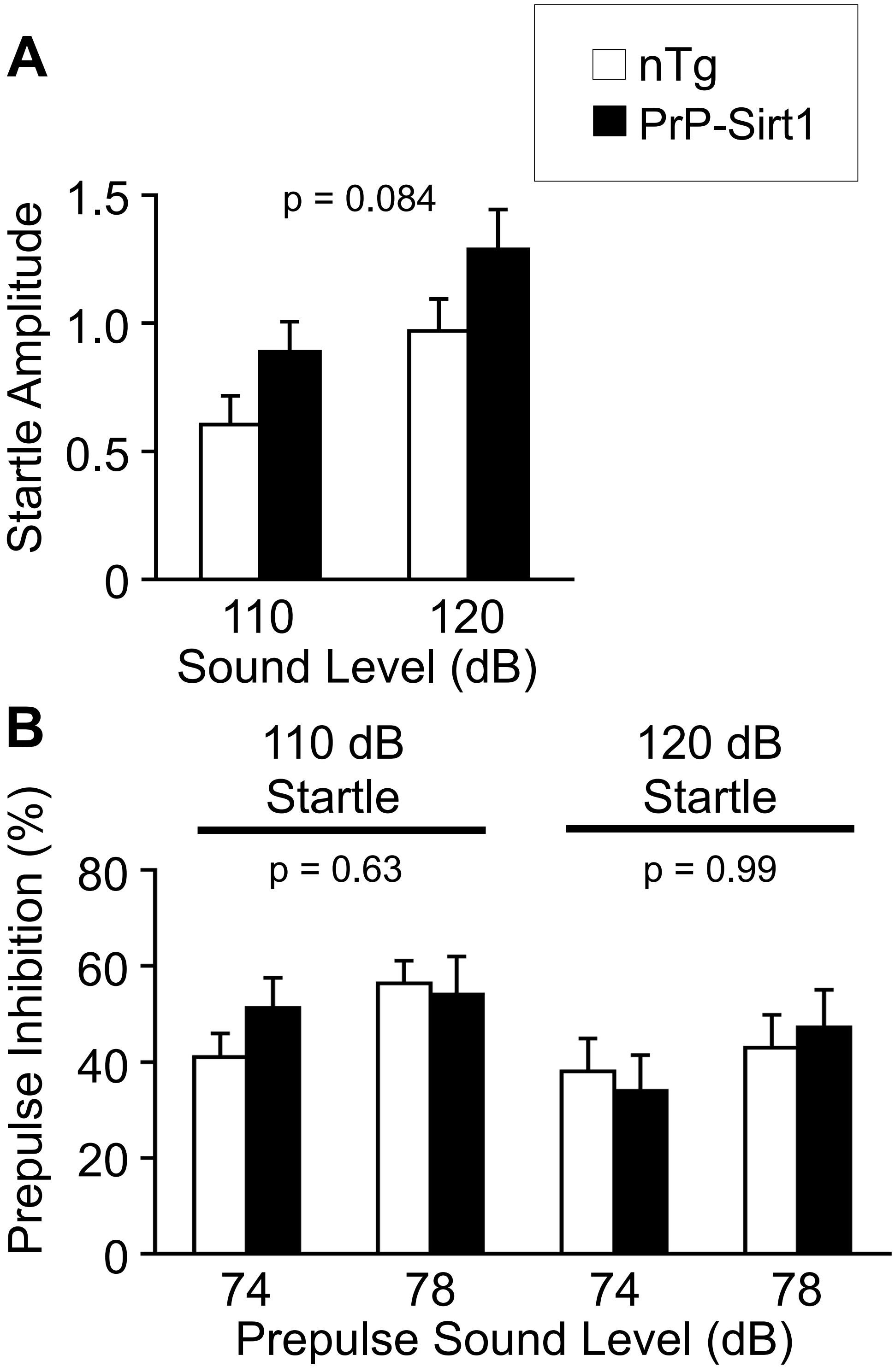

Additional Figure A8

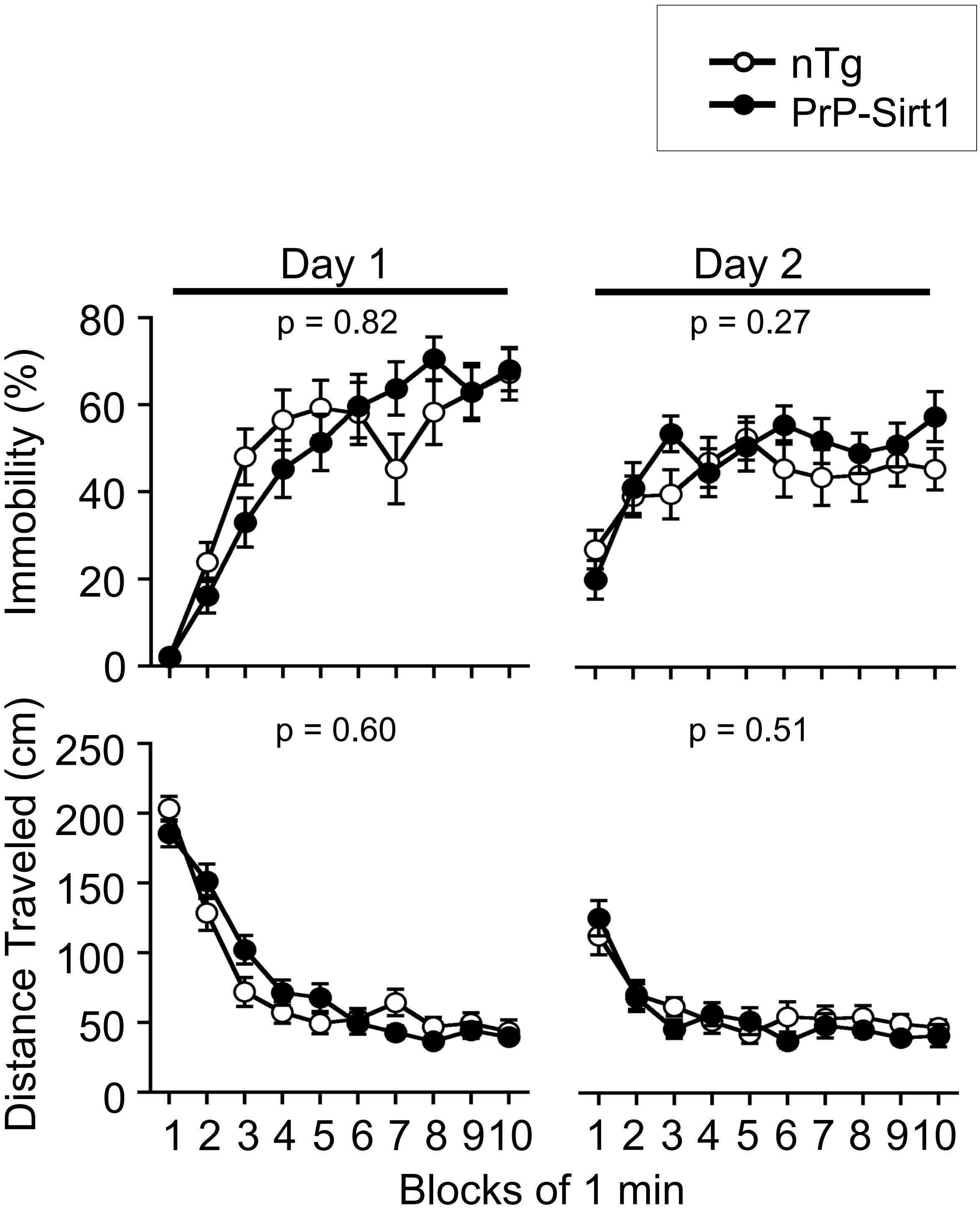

Additional Figure A9

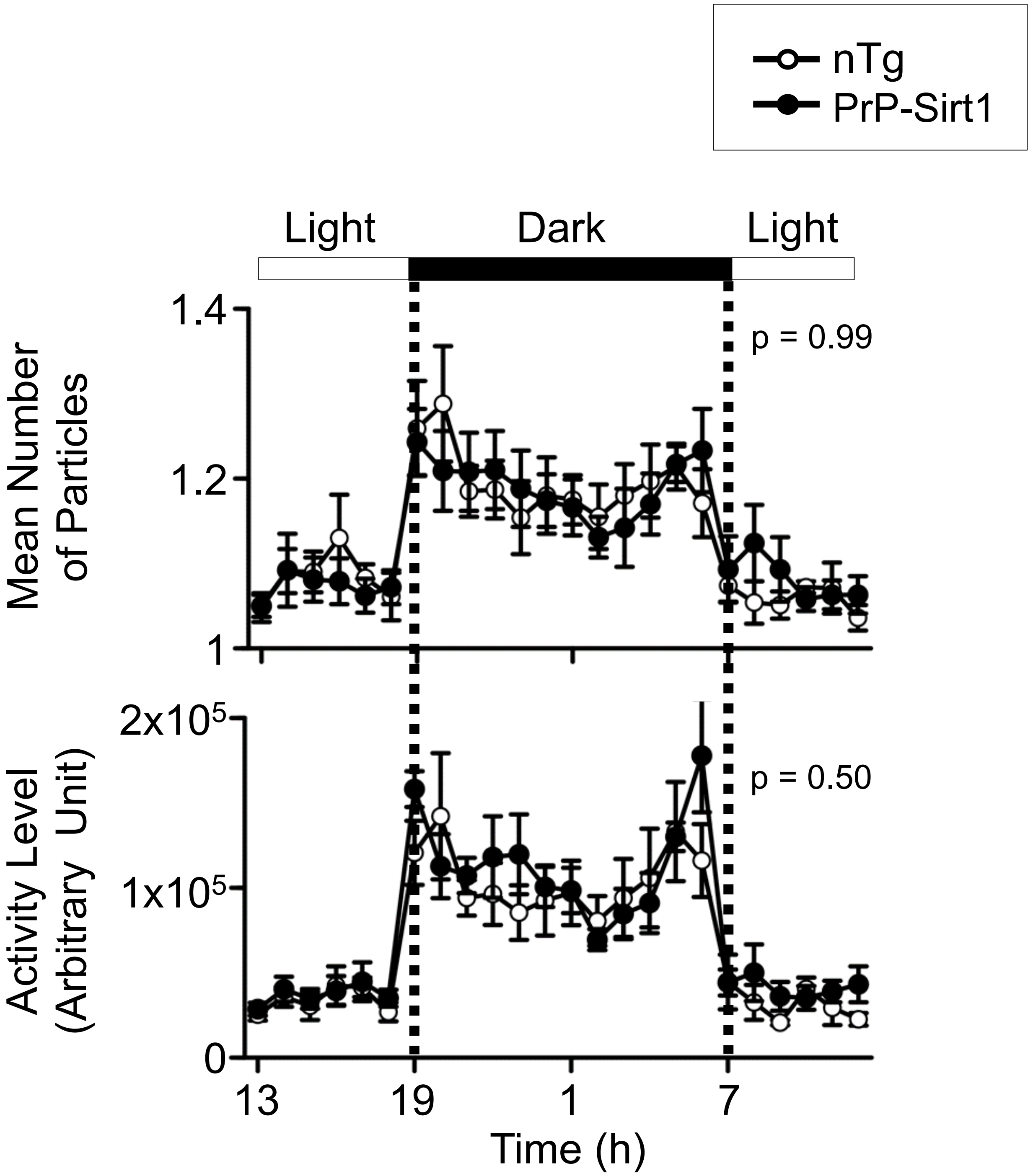

Additional Figure A10

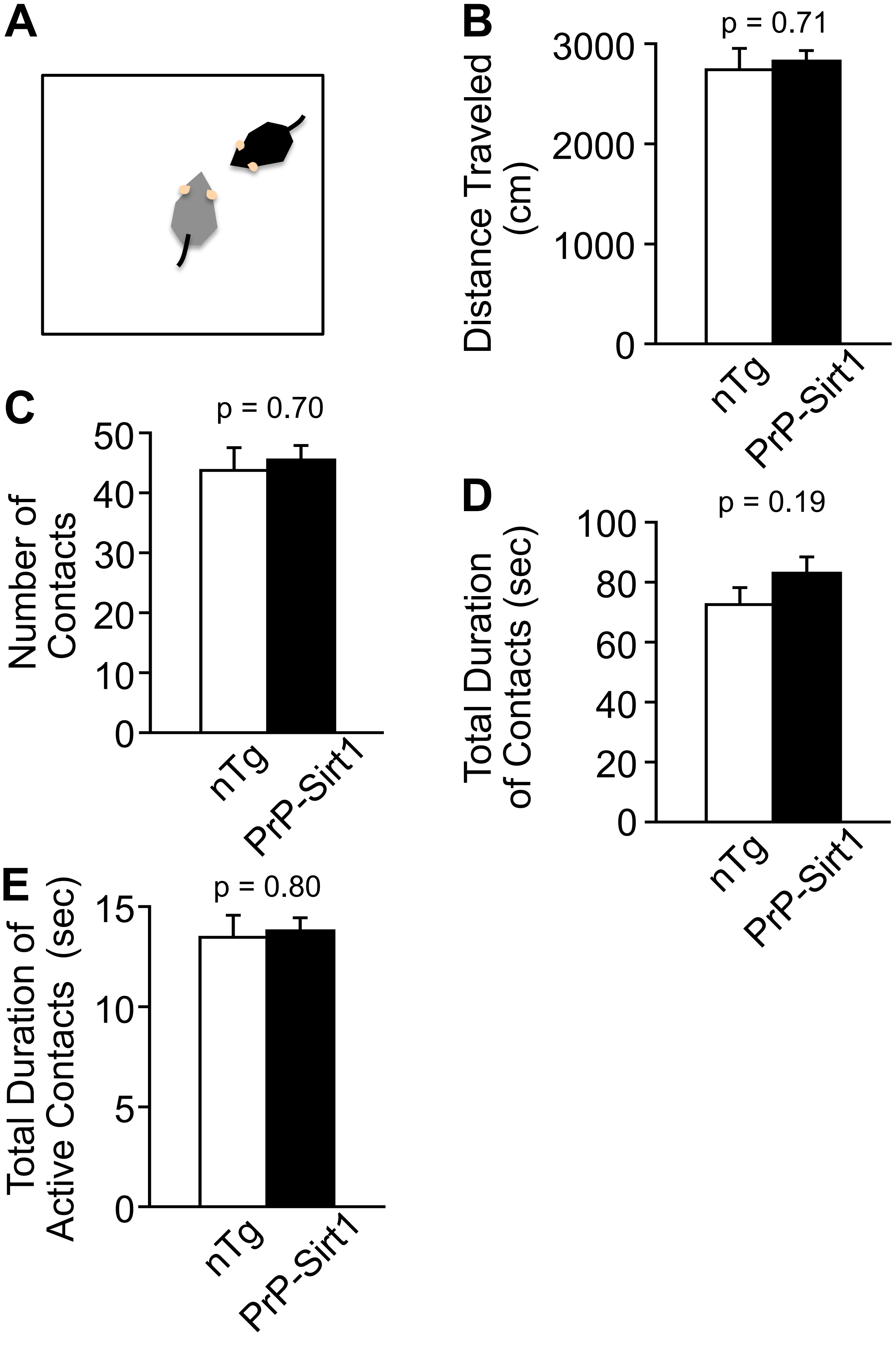

Additional Figure A11

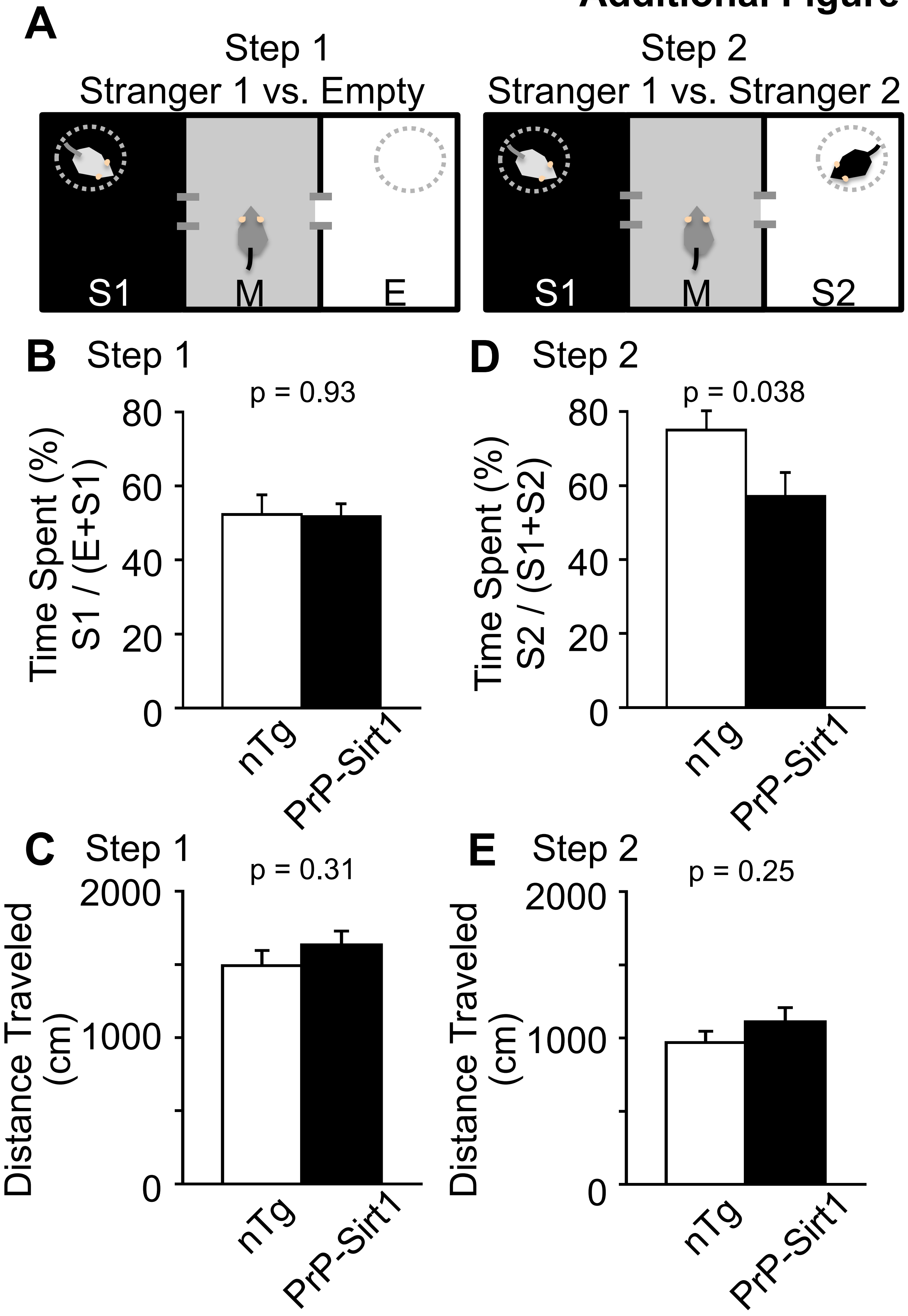

Additional Figure A12

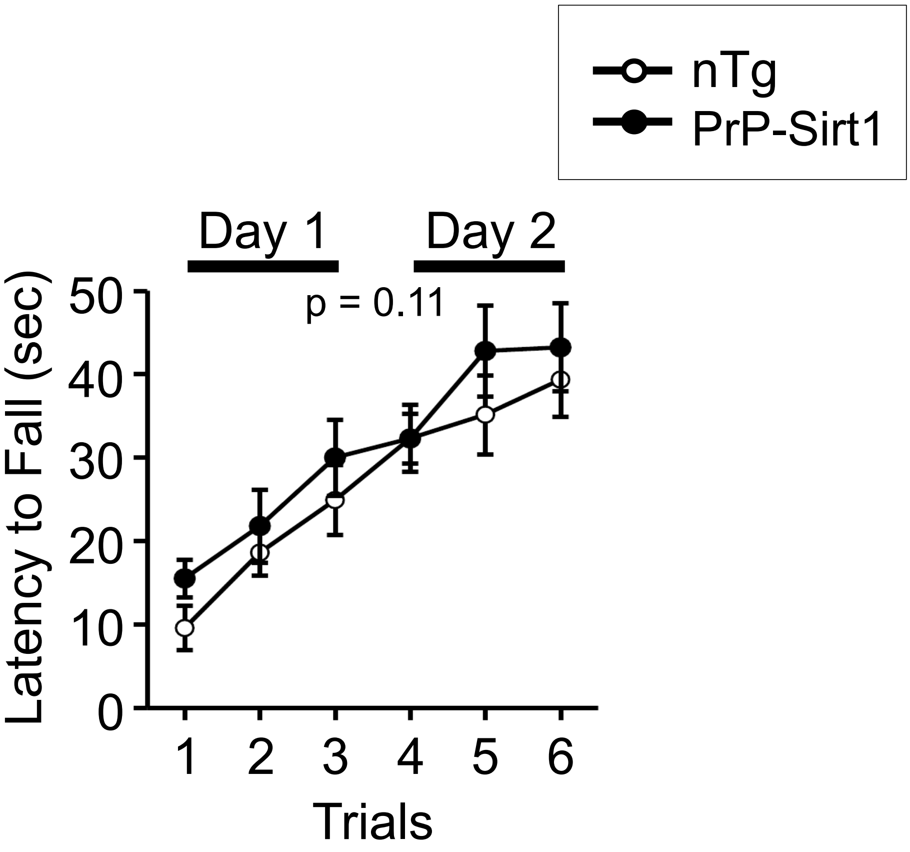

Additional Figure A13

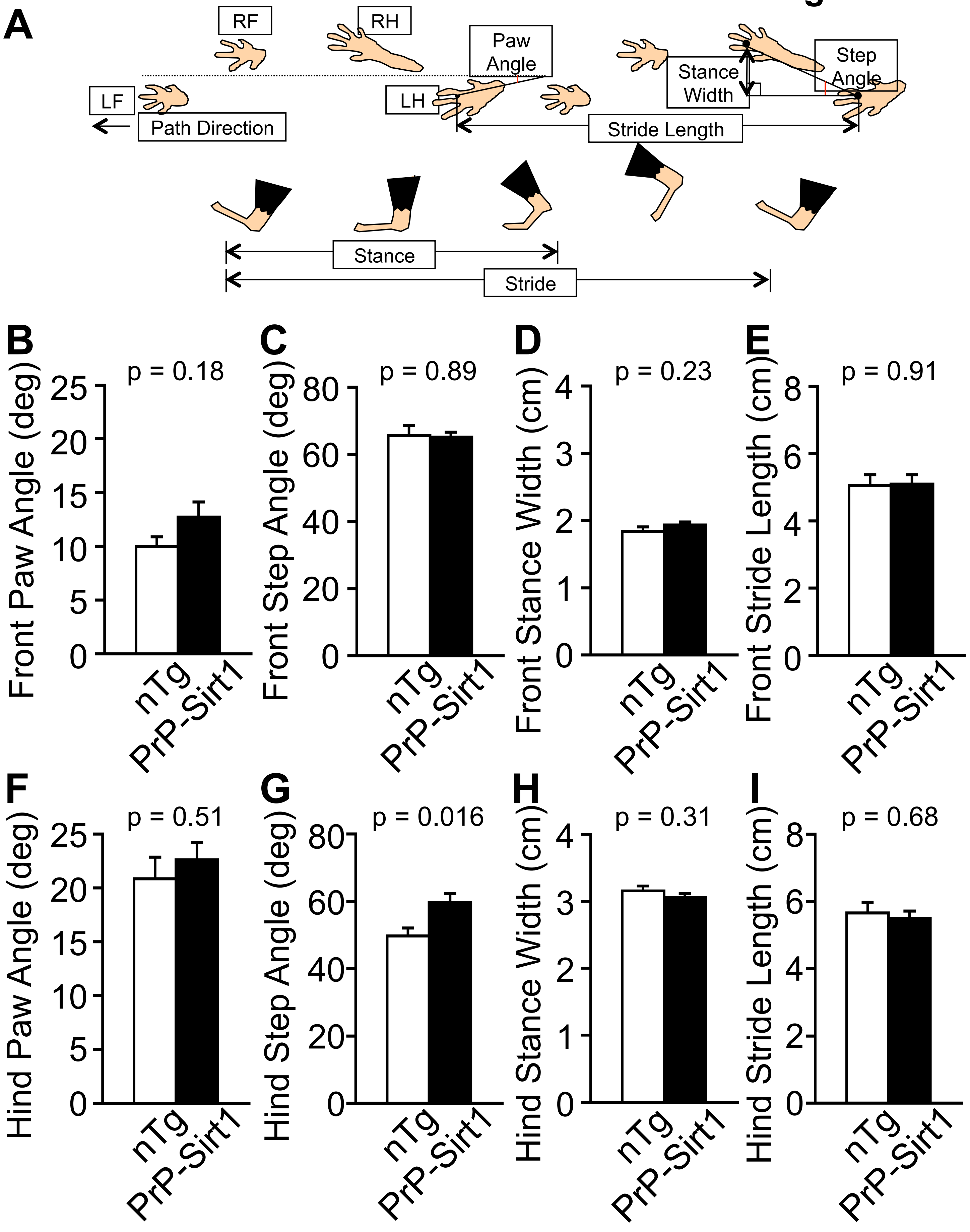

Additional Figure A14

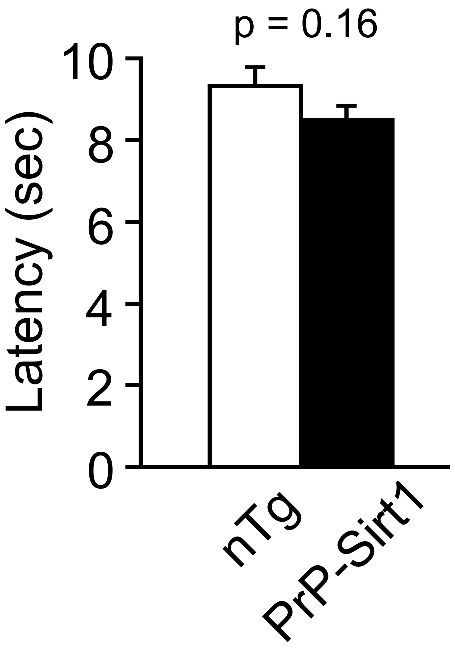

Additional Figure A15

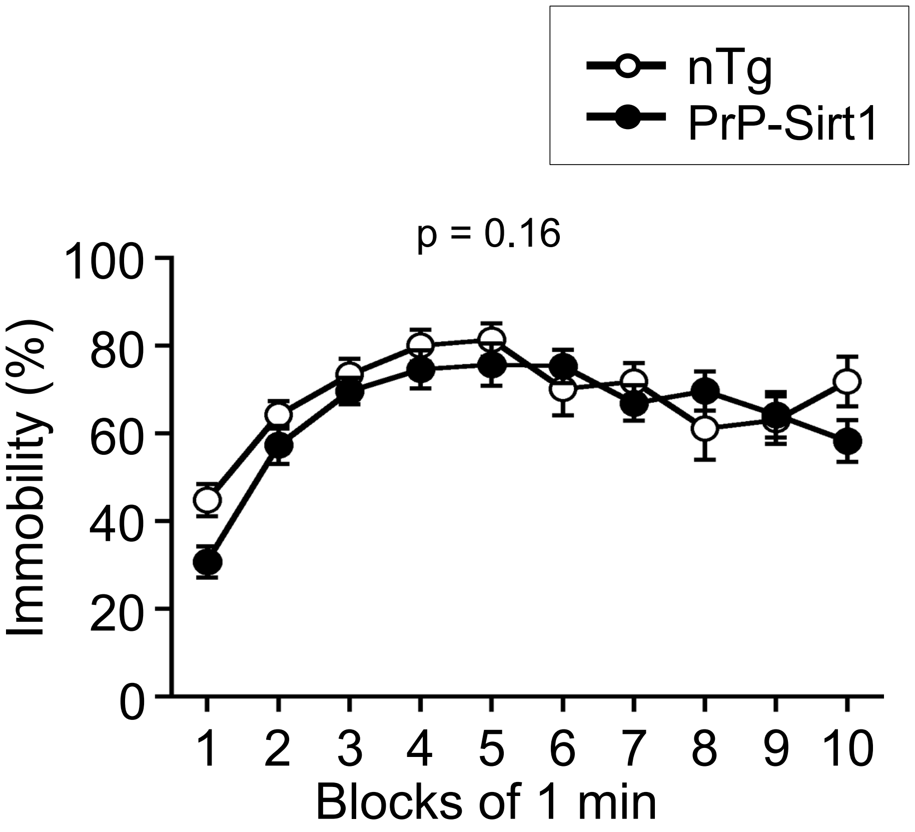

Additional Figure A16

A

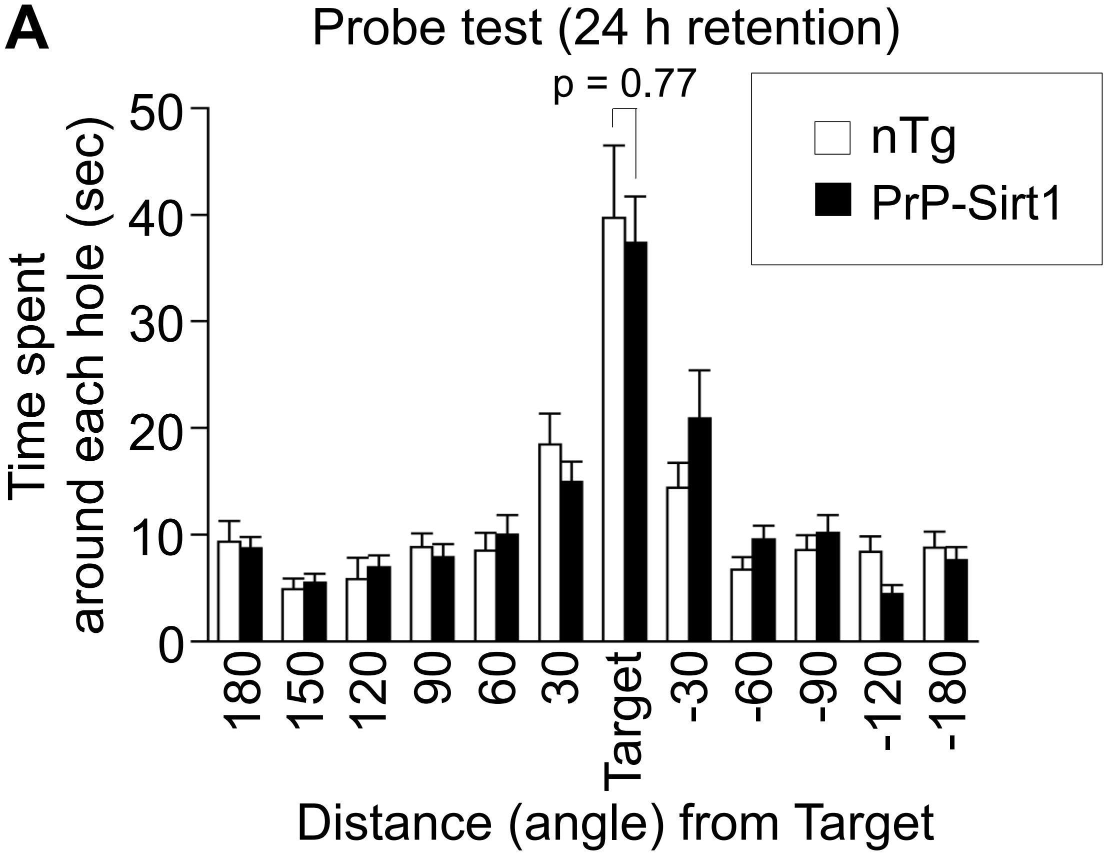

B

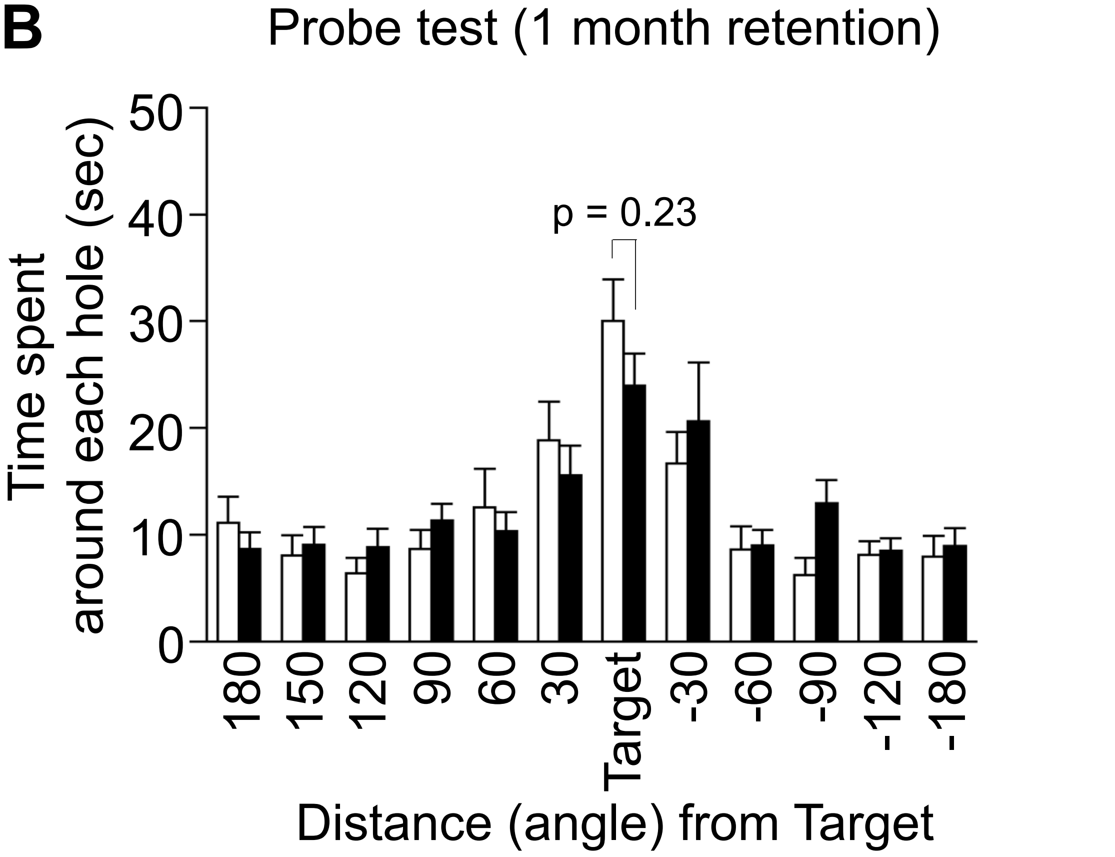

Additional Figure A17

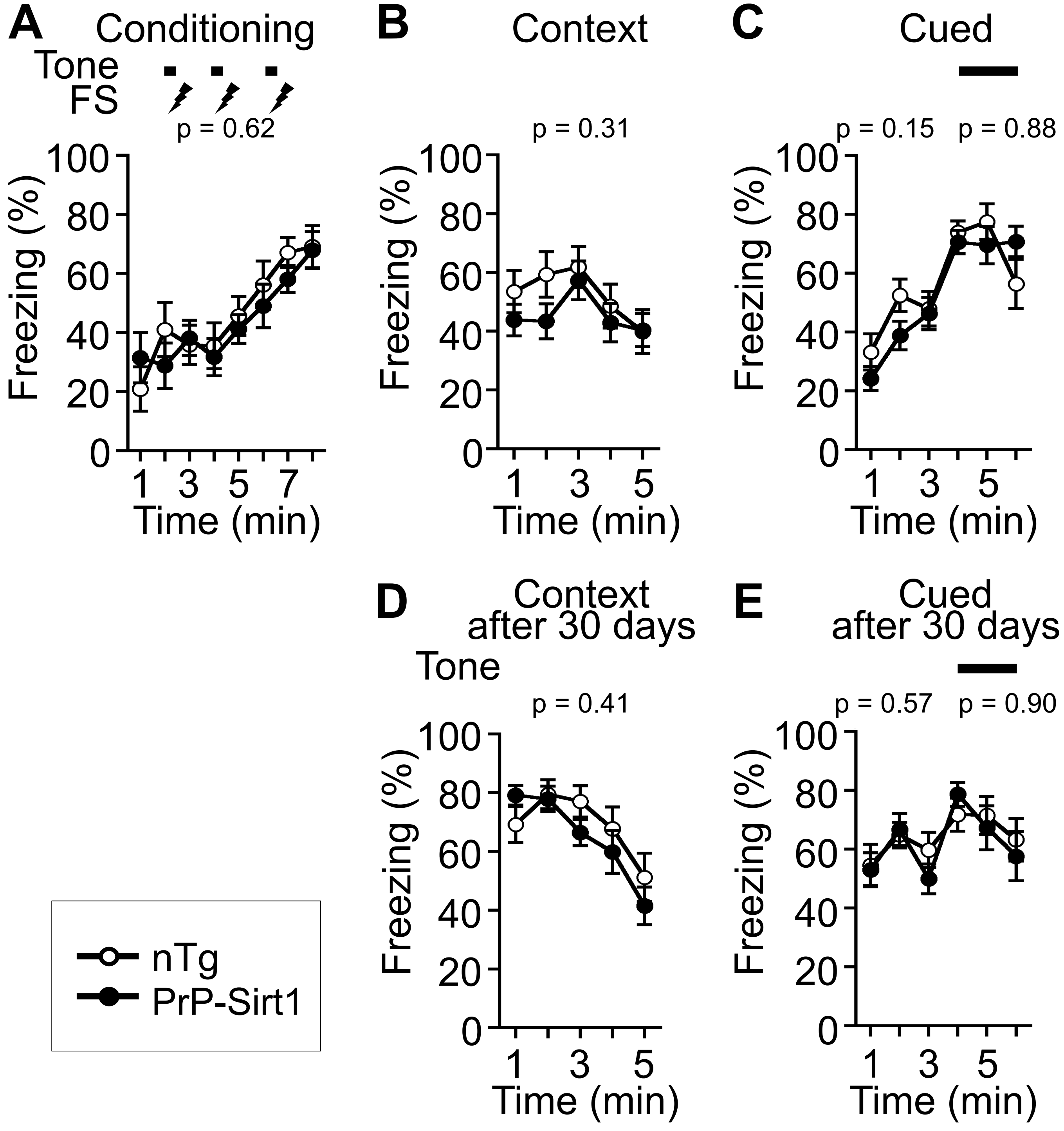

Supplement: Additional file 1 — Additional experimental procedures and figures. [file s13041-014-0062-1-S1.pdf]
